# Supplementary material for: Current regulatory requirements for assessment of immunogenicity for gene therapy medicinal products
Source: Cell Rep Med. 2025 Oct 22;6(12):102422. doi: 10.1016/j.xcrm.2025.102422 (PMC12765835; doi:10.1016/j.xcrm.2025.102422)
Supplement: Document S1. Tables S1 and S2 [file mmc1.pdf]

Cell Reports Medicine, Volume *6*

Supplemental information

Current regulatory requirements for assessment of immunogenicity for gene therapy medicinal products

Christopher J. Mann, Jon Giblin, Manuela Braun, Felicitas Schmid, Maria Rathmann Sørensen, Paolo Caferra, Anett Hudák, Tamás Letoha, Núria Coderch, Timothy P. Hickling, and Mimoun Azzouz

Table S1: Summary of current international regulatory guidelines relevant to the assessment of immunogenicity and immunomodulatory regimens for gene therapy medicinal products (GTMPs)

Region (Issuing Agency)	Guideline / Standard	Manufacturing	Nonclinical					Clinical			General		
		Immunogenicity	Animal models	Alternative models	Pre-existing immunity	Treatment responses	Immunomodulation	Pre-existing immunity	Treatment responses	Immunomodulation	Bioanalytical method and CDx	Paediatric	Other
EU / UK (EMA)	Guideline on quality, non-clinical and clinical requirements for investigational advanced therapy medicinal products in clinical trials (EMA/CAT/22473/2025)	-	+	+/-	-	+/-	-	+/-	+/-	-	-	-	-
EU / UK (EMA)	Guideline on the quality, non-clinical and clinical aspects of gene therapy medicinal products (EMA/CAT/80183/2014)	-	+	-	+	+	-	+	+	+/-	-	-	+[1]
EU / UK (EMA)	Reflection paper on quality, non-clinical and clinical issues related to the development of recombinant adeno-associated viral vectors (EMEA/CHMP/GTWP/587488/2007 Rev. 1)	-	+	-	-	-	-	+	+	-	-	-	-
EU / UK (EMA)	Guideline on safety and efficacy follow-up - risk management of advanced therapy medicinal products (Draft) (EMEA/149995/2008 rev.1)	-	-	-	-	-	-	-	+/-	-	-		+[1, 2]
EU / UK (EMA)	Guideline on the risk-based approach according to annex I, part IV of Directive 2001/83/EC applied to Advanced therapy medicinal products	-	-	-	-	+/-	-	-	+/-	-	-	-	+/-[3]

Region (Issuing Agency)	Guideline / Standard	Manufacturing	Nonclinical					Clinical			General		
		Immunogenicity	Animal models	Alternative models	Pre-existing immunity	Treatment responses	Immunomodulation	Pre-existing immunity	Treatment responses	Immunomodulation	Bioanalytical method and CDx	Paediatric	Other
	(EMA/CAT/CPWP/686637 /2011)												
EU / UK (EMA)	Guideline on quality, non-clinical and clinical aspects of medicinal products containing genetically modified cells (EMA/CAT/GTWP/671639 /2008 Rev. 1 – corr)	-	+	+/-	-	-	-	-	+	-	-	-	+/-[1]
EU / UK (EMA)	Guideline on development and manufacture of lentiviral vectors (CHMP/BWP/2458/03)	-	-	-	-	-	-	-	-	-	-	-	-
EU / UK (EMA)	Guidelines on Good Clinical Practice specific to Advanced Therapy Medicinal Products (C(2019) 7140 final)	-	-	-	-	-	-	+/-	+/-	+/-	-	+/-	+/-[4]
USA (FDA)	Chemistry, Manufacturing, and Control (CMC) information for human gene therapy Investigational New Drug Applications (INDs)	-	-	-	-	-	-	-	-	-	-	-	-
USA (FDA)	Long Term Follow-Up after administration of human gene therapy products	-	-	-	-	-	-	-	+/-	+/-	-	-	-
USA (FDA)	Human gene therapy for rare diseases	-	+/-	-	-	-	-	-	+/-	-	+/-	-	-
USA (FDA)	Clinical considerations for therapeutic cancer vaccines	-	-	-	-	-	-	-	+	+	+	-	+[5]

Region (Issuing Agency)	Guideline / Standard	Manufacturing	Nonclinical					Clinical			General		
		Immunogenicity	Animal models	Alternative models	Pre-existing immunity	Treatment responses	Immunomodulation	Pre-existing immunity	Treatment responses	Immunomodulation	Bioanalytical method and CDx	Paediatric	Other
USA (FDA)	Design and analysis of shedding studies for virus or bacteria-based gene therapy and oncolytic products	-	-	-	-	+/-	-	-	-	+/-	-	-	+/-
USA (FDA)	Considerations for the design of early-phase clinical trials of cellular and gene therapy products	-	-	-	-	+/-	-	+	+	+	-	+	+/-[1]
USA (FDA)	Human gene therapy products incorporating human genome editing	-	-	-	-	+	-	+/-	-	-	-	+	-
USA (FDA)	Preclinical assessment of investigational cellular and gene therapy products		+	+	-	+/-	+/-	-	-	-	-	-	-
USA (FDA)	Frequently asked questions - developing potential cellular and gene therapy products	-	+	-	-	-	-	-	-	-	-	-	-
Japan (PMDA)	PMDA (Japan): ensuring the quality and safety of gene therapy products, 9 July, 2019	-	-	-	-	+/-	-	-	+/-	-	-	-	-
Japan (PMDA)	White-paper for quality and safety for gene therapy products using gene editing technology	-	-	-	-	+	-	-	+	-	-	-	+/-[1]

Key concepts related to immunogenicity and immunomodulation that were identified as being the most relevant in the landscape assessment are shown at the top. Each guideline is qualified for relative coverage of these key areas with “-“ indication no or negligible coverage, “+/-“ some coverage and “+” indication that the key area is addressed.

[1] Refers to risk of autoimmunity (including possibly reference to cross-reactivity or bystander autoimmune responses).

[2] Specific reference to risk management plan (RMP).

[3] Discussion on localisation of relevant studies and assessment in regulatory documentation.

[4] Discussion of specific pharmacovigilance aspects.

[5] Discussion on use of adjuvants in addition to therapeutic agent (and independently of other immunomodulatory agents)

Table S2: List of additional guidelines and monographs not specific to GTMPs but relevant to evaluation of immunogenicity to therapeutic proteins and biologics and/or developing companion diagnostics (CDx)

**Biologic product guidance**	
EU /EEA	EMA Guideline on Immunogenicity assessment of therapeutic proteins, Rev 1, 2017 (EMEA/CHMP/BMWP/14327/2006 Rev 1)
USA	FDA Immunogenicity Assessment for Therapeutic Protein Products, 2104
USA	FDA Immunogenicity Testing of Therapeutic Protein Products —Developing and Validating Assays for Anti-Drug Antibody Detection, 2019
**Guidance/legislation relevant to bioanalytical methods, companion diagnostics and/or patient stratification**	
EU /EEA	Regulation (EU) 2017/746 of the European parliament and of the council of 5 April 2017 on in vitro diagnostic medical devices and repealing Directive 98/79/EC and Commission Decision 2010/227/EU
USA	FDA In Vitro Companion Diagnostic Devices, 2014
Other / Rest of the world (RoW)	ICH M10 Bioanalytical method validation and study sample analysis<br>CLSI EP24-A2 Assessment of the Diagnostic Accuracy of Laboratory Tests Using Receiver Operating Characteristic Curves; Approved Guideline – Second Edition<br>CLSI EP28-A3c Defining, Establishing, and Verifying Reference Intervals in the Clinical Laboratory; Approved Guideline – Third Edition<br>CLSI EP12-A2 User Protocol for Evaluation of Qualitative Test Performance; Approved Guideline – Second Edition<br>CLSI EP17-A Protocols for Determination of Limits of Detection and Limits of Quantitation. Approved Guideline<br>CLSI EP17-A2 Evaluation of Detection Capability for Clinical Laboratory Measurement Procedures; Approved Guideline – Second Edition
International Standards Organisation (ISO)	MD ISOs for combined ATMPs - ISO/TS 10993-20:2006(en) Biological evaluation of medical devices — Part 20: Principles and methods for immunotoxicology testing of medical devices
Key papers	Evaluation of Cellular Immune Response to Adeno-Associated Virus-Based Gene Therapy (Gorovits *et al*., 2023)<br>Evaluation of the Humoral Response to Adeno-Associated Virus-Based Gene Therapy Modalities Using Total Antibody Assays (Gorovits *et al*., 2021)<br>Bioanalysis of adeno-associated virus gene therapy therapeutics: regulatory expectations (Gorovits *et al*., 2020)<br>2023 White Paper on Recent Issues in Bioanalysis: ISR for ADA Assays, the Rise of dPCR vs qPCR, International Reference Standards for Vaccine Assays, Anti-AAV TAb Post-Dose Assessment, NanoString Validation, ELISpot as Gold Standard (Part 3 - Recommendations on Gene Therapy, Cell Therapy, Vaccines Immunogenicity & Technologies; Biotherapeutics Immunogenicity & Risk Assessment; ADA/NAb Assay/Reporting Harmonization (Mora et al., 2024)<br>2022 White Paper on Recent Issues in Bioanalysis: FDA Draft Guidance on Immunogenicity Information in Prescription Drug Labeling, LNP & Viral Vectors Therapeutics/Vaccines Immunogenicity, Prolongation Effect, ADA Affinity, Risk-based Approaches, NGS, qPCR, ddPCR Assays (Part 3 - Recommendations on Gene Therapy, Cell Therapy, Vaccines Immunogenicity & Technologies; Immunogenicity & Risk Assessment of Biotherapeutics and Novel Modalities; NAb Assays Integrated Approach) (Pan et al., 2023)

Definitions:

Autoimmunity: an immune response against an individual's own cells, tissues, or components, potentially leading to disease.

Bystander activation: antigen-independent activation of self-reactive B and T cells that may promote autoimmunity.

Companion diagnostic (CDx): an in vitro diagnostic test that supports the safe and effective use of a specific corresponding medicinal product, for example, by identifying patients that are suitable or unsuitable for treatment such as those with critical levels of pre-existing immunity (Regulation 2017/746/EU).

Cross-reactivity: when an antigen elicits immune responses that cross react with other antigens.

Immunogenicity: ability of an agent to generate immune response against itself.

Immunomodulation: regulation and modulation of immunity by agents that enhance or suppress immunological function.

Pharmacovigilance: the science and activities relating to the detection, assessment, understanding, and prevention of adverse effects or any other drug-related problem.
