# Supplementary material for: Current regulatory requirements for assessment of immunogenicity for gene therapy medicinal products
Source: Cell Rep Med. 2025 Oct 22;6(12):102422. doi: 10.1016/j.xcrm.2025.102422 (PMC12765835; doi:10.1016/j.xcrm.2025.102422)
Supplement: Document S2. Article plus supplemental information [file mmc2.pdf]

Cell Reports Medicine

CellPress
OPEN ACCESS

Review

Current regulatory requirements for assessment of immunogenicity for gene therapy medicinal products

Christopher J. Mann,[1,*] Jon Giblin,[1] Manuela Braun,[2] Felicitas Schmid,[2] Maria Rathmann Sørensen,[3] Paolo Caferra,[4] Anett Hudák,[5] Tamás Letoha,[5] Núria Coderch,[1] Timothy P. Hickling,[6] and Mimoun Azzouz[7,8,*]

[1]Asphalion SL, Barcelona, Spain
[2]Bayer AG, Wuppertal, Germany
[3]Novo Nordisk A/S, Copenhagen, Denmark
[4]Sanofi, Research & Development, Sanofi, Amsterdam, the Netherlands
[5]Pharmacoidea Ltd., Szeged, Hungary
[6]Roche Innovation Centre Welwyn, Pharma Research and Early Development, Roche, Welwyn Garden City, UK
[7]Sheffield Institute for Translational Neuroscience, Division of Neuroscience, School of Medicine and Population Health, University of Sheffield, Sheffield, UK
[8]Gene Therapy Innovation & Manufacturing Centre (GTIMC), University of Sheffield, Sheffield, UK
*Correspondence: cmann@asphalion.com (C.J.M.), m.azzouz@sheffield.ac.uk (M.A.)
https://doi.org/10.1016/j.xcrm.2025.102422

SUMMARY

Gene therapy medicinal products (GTMPs) are currently undergoing intense industrial expansion and technological advancement. However, one issue facing development of most GTMPs is the generation of unwanted immune responses. Immunomodulatory strategies are also often applied in conjunction with GTMP administration to suppress or enhance these responses. This review focusses on the global regulatory requirements for immunogenicity assessments and immunomodulation in relation to GTMPs. The specific aims are to (1) identify the principal international guidelines; (2) identify areas of concordance and discrepancy between guidelines; (3) propose areas where guidelines could be harmonized; and (4) predict areas, which future guidance may address. Methodologies used included surveillance of literature, international guidelines, advocacy initiatives, and compilation of previous regulatory advice received. Overall, there is a clear absence of and need for GTMP-specific guidance on immunogenicity and immunomodulation. Several specific measures and areas for future regulatory harmonization and coverage are proposed.

INTRODUCTION

Advanced therapy medicinal products (ATMPs), including gene therapy medicinal products (GTMPs), cell therapies, and tissue engineered products, represent a major paradigm shift in modern medicine. ATMPs are currently undergoing intense scientific, medical, and industry interest.[1] This is driven partly by the broader promises of such technologies where a single administration may be sufficient to mediate long-term benefits for rare, serious, or life-threatening conditions. However, the growing diversity and innovation of the underlying technologies challenge the regulators to keep pace. Regulatory delays often stem from the inherently reactive nature of oversight bodies until empirical evidence accumulates and stakeholder interaction and consensus grow. For ATMPs, the pace of legal and ethical reviews to safeguard public interest requires special consideration, as does the challenge of adapting traditional small-molecule drug development regulatory frameworks. Consequently, there are occasionally regulatory gaps or lags in available guideline. In addition, as many ATMPs emerge from academia,[2,3] regulatory experience may be limited with consequences for efficient translation to the clinic. As ATMPs often demand high prices,[4] opportunities to accelerate translational development and reduce development costs and regulatory burdens, while increasing patient availability, are important goals.

In 2007, the legislative framework for the development and authorization of ATMPs in the European Union (EU) was established with the implementation of Regulation 1394/2007/EC. Since then, 28 ATMPs have been approved in the EU (as of May 2025), after a positive evaluation by the European Medicines Agency (EMA). In the USA, as of May 2025, there are 45 ATMPs approved by the Food and Drug Administration (FDA).

The ARDAT (Accelerating Research and Development for Advanced Therapies) consortium is a collaboration between academia; micro-, small-, and medium-sized enterprises; and European Federation of Pharmaceutical Industries and Associations (EFPIA) members. ARDAT is supported financially by the Innovative Health Initiative and EFPIA. ARDAT aims to fill current ATMP knowledge gaps in several areas, including immunology. Specific ARDAT objectives include identifying areas for

© 2025 The Authors. Published by Elsevier Inc.
This is an open access article under the CC BY license (http://creativecommons.org/licenses/by/4.0/).

regulatory harmonization; developing improved, standardized models for predicting immunogenicity in humans; and understanding the clinical factors and immunomodulatory interventions related to patient clinical access.[5] In line with this, the objective of this review was to perform an evaluation of the current regulatory requirements for immunogenicity assessment and use of immunomodulation relevant for global development of GTMPs. The specific aims were to (1) identify the principal international guidelines; (2) identify areas of concordance and discrepancy between the guidelines; (3) propose areas where these guidelines could be harmonized; and (4) predict areas, which future guidance may address. This is thus a regulatory assessment and not a technical review of GTMP immunity, which has been extensively covered elsewhere. Neither does the review cover details of all immunomodulatory agents, but it rather considers the regulatory implications of their use in conjunction with GTMPs. Finally, the scope of the review is also limited to GTMPs as currently defined in the EU.

For the purposes of this review, immunogenicity is defined as the ability of an agent to generate immune response against itself or its transgene product. Immunomodulation is defined as modulation of immunity by agents that enhance or suppress immunological function.

Generation of an unwanted immune response is a major issue facing clinical development of most GTMPs. Immune responses can be triggered by viral capsid proteins, impurities, or the transgene product.[6–9] Immunogenicity has also been reported against cell-based GTMPs such as genetically modified cells and gene editing components.[10–12] Immune responses usually involve both innate immunity and adaptive immunity. Pre-existing immunity may limit access of patients to clinical trials or approved treatment.[6,7] Immunogenicity could potentially have important consequences including loss of efficacy over time due to immune-mediated clearance or safety concerns such as immune-related adverse events (irAEs), hepatitis, and even death.[13–15] Importantly, animal models for studying immunity have limited predictive value prior to clinical assessment,[8] and new models and tools are needed for predicting and characterizing safety concerns related to unwanted immunity. Most "classic" GTMPs adopt a gene replacement approach, for which immune responses are not desirable. However, there is a subclass of GTMPs where an immune response is the intended effect. This subclass includes the so-called "therapeutic vaccines" for the treatment of cancer[16] or chronic infectious disease.[17] Finally, several immunomodulatory strategies, some of which are investigational, are also often applied in conjunction with GTMP administration.[5,18–20] These immunomodulation strategies may be intended to either suppress or enhance the immune response. Immunogenicity assessments and application of immunomodulation may also be a specific pharmacovigilance, post-marketing, and/or long-term follow-up (LTFU) requirement linked to the overall risk management of the product.

The methodology used for this review included identifying and evaluating the key international regulatory guidelines and standards related to GTMP immunogenicity and immunomodulation that were available at the time of publishing. Specific regulatory topics were identified and supplemented by examples from the literature and the public domain, including other advocacy initiatives.[21] In addition, ARDAT partners also revised and compiled any related confidential regulatory advice received to help identify key topics. The review is intended to be relevant for all types of GTMPs, but a primary focus is on viral vectors and especially adeno-associated virus (AAV)-based vectors as these are the most widely used and successful clinical vectors with several examples already approved by EMA and FDA. For reference, GTMPs are considered to include both viral and nonviral approaches involving therapeutic nucleic acid sequences and include gene-modified cells, gene editing, oncolytic viruses, and therapeutic vaccines (but not prophylactic vaccines for infectious disease).

CURRENT REGULATORY REQUIREMENTS

The current regulatory guidelines relevant to immunogenicity assessments of GTMPs are listed in Table S1 and cover the EU (including the UK), USA, and Japan. Some guidelines referring to specific clinical indications (e.g., hemophilia) may be referred to in the text, but only broadly applicable GTMP guidelines are included in Table S1. Additional monographs and regulatory documents related to immunogenicity but not specific to GTMPs are listed in Table S2, including those specific to companion diagnostics (CDx), as well as therapeutic protein and biological product immunogenicity guidelines.

Key topics related to GTMP immunogenicity and immunomodulation that were considered the most relevant were identified first. Key topics included: product design, manufacturing, animal models (including potential alternatives), pre-existing immunity and treatment responses (nonclinical and clinical), immunomodulation regimens, bioanalytical methods, and pharmacovigilance requirements. Subsequently, guidelines were assessed to qualify whether they covered these topics specifically (+), indirectly (±), or not at all (−) (Table S1). Gaps in the existing regulatory guidance can thus be seen where some of these key areas are not covered. For example, there is currently limited coverage of the requirements for performing immunomodulation in nonclinical models, except for indirect reference in a single FDA guideline (Table S1). The specific regulatory issues identified for each of the key topics are further summarized in Table 1 and Figure 1 and detailed in separate sections in the following.

Overall, while EMA and FDA have published regulatory guidance with specific recommendations for the assessment of immunogenicity of therapeutic proteins and several white papers are available (Table S2),[22–24] the applicability of these recommendations for GTMP immunogenicity needs to be reevaluated. The emphasis of these guidelines is largely on the consequences of humoral immune responses for safety and efficacy, focusing on the measurement of total anti-drug antibodies followed by neutralizing antibodies (nAbs) in a tiered approach. Such a tiered approach may not be the most appropriate in the context of GTMPs since an immune response is generally always expected and observed. Moreover, these protein-specific guidelines provide few recommendations for assessments of cellular responses, which play a much larger role for GTMPs than for therapeutic proteins in

CellPress
OPEN ACCESS

Table 1. Summary of key concept areas and specific regulatory considerations for immunogenicity and immunomodulation assessments during development of gene therapy medicinal products

Key concept area	Specific considerations
Product design	• product design elements to reduce immunogenicity (e.g., CpG removal, humanization)<br>• representativeness of material(s) used during early development to clinical material (including use of surrogate products, e.g., species homologous transgenes)<br>• impact of changes in product design during development
Manufacturing	• product-related impurities: empty:full capsids, non-infectious particles, replication competent virus (RCV), aggregates<br>• process-related impurities: host cell protein (HCP), host cell DNA, residual plasmid DNA, other impurities (e.g., nucleases and other proteins)<br>• immunogenicity of excipients
Nonclinical	• species specificity of immune response (species-specific product components; animal models may not predict human response)<br>• limited predictivity of current nonclinical models<br>• acceptability of novel approaches to predicting immunogenicity and safety concerns
Nonclinical/clinical	• pre-existing immunity<br>• treatment response involving innate (including complement), humoral, and cellular immunity<br>• immune response to transgene product<br>• immunomodulator requirements
Clinical	• developmental differences in immune system between pediatric patients and adults<br>• immune-related adverse events (irAEs)<br>• immune status of patient (disease state influence)<br>• autoimmunity
Bioanalytical methods	• companion diagnostic (CDx) development for patient selection based on pre-existing immunity<br>• choice of analytes and assay methods for immunogenicity assessment<br>• validation parameters and method sensitivity<br>• comparability of methods between sponsors (harmonization)
Pharmacovigilance	• long-term follow-up (LTFU) and risk management<br>• safety reporting including possible interactions

relation to the potential for adverse reactions and loss of efficacy. Therefore, specific guidance for GTMPs would be welcome. This guidance would complement the broader diagnostic guidelines and standards applicable to assessment of pre-existing antibodies to GTMPs for the purpose of patient selection/stratification (e.g., Clinical & Laboratory Standards Institute guidelines) (Table S2).

One specific area where there is limited guidance and no clear product regulatory definitions is the “therapeutic vaccine” subclass of GTMPs. These types of products are not evaluated as standard prophylactic vaccines. FDA has published one guideline that focusses primarily on clinical aspects (Table S1). Further reference to this GTMP subclass is made in the following sections, especially where current regulatory requirements diverge from “classic” GTMPs.

PRODUCT DESIGN ASPECTS

Viral vectors contain a recombinant vector genome (i.e., the expression cassette) surrounded by a protein capsid and sometimes an envelope. All parts of the vector can be immunogenic, including adaptive immune responses to capsid proteins or stimulation of the innate immune system by nucleic acid (vector-derived or impurities).[18,25–27] Innate responses to charged DNA and RNA such as oligonucleotides have been reported. Immune responses have also been detected to some nonviral carriers such as lipid nanoparticles.[28,29] Immune responses to scaffolds and other biomaterials that may be used with combined GTMPs are an important consideration but beyond the scope of this review (however, relevant ISO guidelines are referenced in Table S2). Such materials are more frequently used for cell- and tissue-based ATMPs, but some approaches have tested local administration of AAV with alginate hydrogels, for example Remes et al.[30]

Various strategies to reduce aspects of unwanted GTMP immunogenicity have been proposed. Some examples include use of CpG-free sequences, inverted terminal repeat (ITR) modifications, use of modified nucleosides in mRNAs, attaching stealth molecules, engineering the capsid, or use of microRNA (miRNA) binding sites to de-target transgene expression from antigen-presenting cells (APCs) or provide tissue specificity, among others.[25,26,31–33] Artificial intelligence (AI) approaches have been recently applied to AAV capsid design to reduce immunogenicity.[34] Changes in product design or composition during development can alter the regulatory definition of the active substance. Significant modifications may create a new active substance, impacting the product’s regulatory status, including toxicity studies or orphan drug designation. Additionally, complexing an existing substance with new materials to reduce immunogenicity can affect pharmacokinetics, requiring new nonclinical studies on dosing, biodistribution, or toxicity. Thus, a risk-based approach to identify immunogenic aspects, or to plan future design modification and comparability testing strategies, should be conducted early in product development.

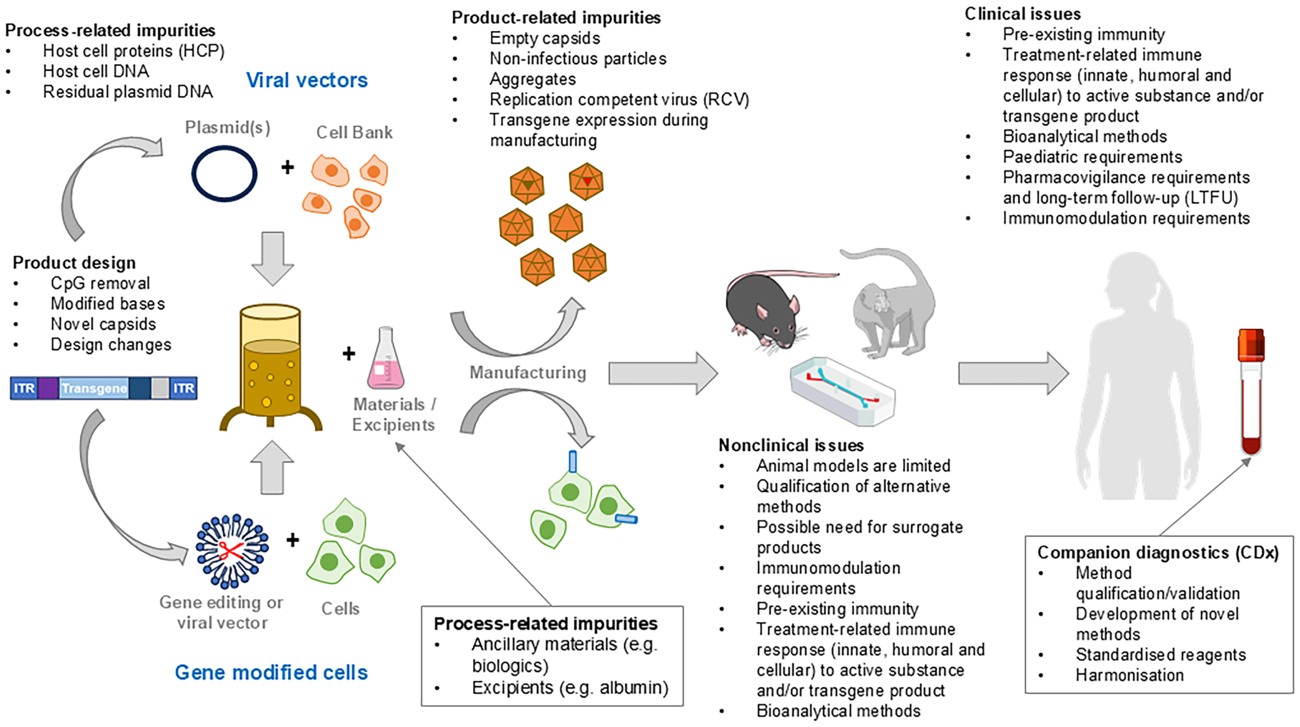

Figure 1. Summary of key regulatory considerations for assessment of immunogenicity and immunomodulation relevant to the global development of gene therapy medicinal products

Illustration includes some elements from NIAID NIH BioArt source (bioart.niaid.nih.gov/bioart).

MANUFACTURING AND IMMUNOGENICITY

A detailed discussion of GTMP manufacturing is beyond the scope of this review.[35,36] However, there are several important areas of manufacturing linked to immunogenicity that are discussed briefly in terms of their regulatory connotations (Table 1).

One of the important manufacturing concerns are product-related impurities that may be immunogenic.[36] Examples include empty capsids and non-infective particles, which have been shown to activate T cells but are currently difficult to remove from most viral manufacturing processes.[6,21,36] To our knowledge, there is currently no formal regulatory recommendation for an acceptable level of empty capsids or non-infectious particles. Additional product-related impurities associated with immunogenicity include post-translational modifications (PTMs) of capsid proteins, including glycosylation, acetylation, phosphorylation, and methylation.[37–39] Importantly, PTMs are not always characterized at early stages of development and are therefore not included as part of release specifications despite the fact that they are known to change depending on the manufacturing platform used.[40] Spontaneous deamidation of asparagine to aspartic acid in capsid proteins can also compromise the performance of ELISpot assays with implications for assay reliability and establishment of reference standards.[38,39] Characterization of PTMs is considered an important emerging area of GTMP manufacturing and is under-addressed in current guidelines.

Likewise, process-related impurities may also include several potential immunogenic components.[36,41,42] One of the most important is host cell protein (HCP) impurities. Most GTMPs are produced with human cells, which should reduce the risks of HCP immunogenicity, but this is not always the case as baculovirus systems are frequently used for AAV manufacture.[40,41] Enveloped viruses such as lentivirus or vesicular stomatitis virus are used as gene therapy vectors and oncolytic viruses, respectively. As enveloped viruses are usually produced in human cells, the natural envelope will contain human proteins, complicating determination of HCPs. Similarly, extracellular vesicles (EVs) are emerging as an important therapeutic approach and are also generally produced in human cells. The composition of EVs will also change depending on cell production system, and, although some immunogenicity risk does exist (especially in animal models when using human cell-derived EVs), it may be modest.[43–45] Finally, many EVs also have an endogenous immunomodulatory function.[46] Overall, there are no reported immune responses to HCP from use of other common biologicals.[47] Nonetheless, there is currently no clear guidance on the accepted levels of HCP in GTMPs, and additional guidance would be welcome.

Host cell nucleic acid and residual plasmid DNA may also activate the innate immune system through CpG recognition and pattern recognition receptors (PRRs).[27] In addition, transcription initiated from promoters on residual packaging plasmids, such as the AAV P5 promoter often used to drive *Rep* gene expression, can also result in production of immunogenic proteins.[48]

Bacterial host cell impurities such as lipopolysaccharide or endotoxin may also activate the innate immune system via Toll-like receptor (TLR)4 signaling.[27]

Immunogenic impurities can include raw and ancillary materials like animal serum and biological reagents (e.g., cell stimulating molecules and insulin). Replication-competent virus (RCV) is a critical quality attribute of viral vector manufacturing. RCV potentially enhances immunogenicity via local amplification. While the FDA provides recommendations regarding levels of replication-competent adenovirus (RCA) (a maximum level of 1 RCA in 3×10^{10} viral particles),[49] no clear limits exist for AAV. Guidance on specific analytical method requirements for RCV determinations would be valuable due to their complexity.

Finally, excipients, particularly recombinant or human proteins, may be immunogenic in animal studies. Diluents should be free from aggregates and/or subvisible particles that may cause local site reactions or enhance immunogenicity.[50]

SAFETY: NONCLINICAL MODELS

Animal models for the study of immune responses to GTMPs have been summarized elsewhere.[51–54] In general, the prediction of immunogenicity and associated toxicities in nonclinical models *in vivo* is limited. For example, a robust cellular immune response to the AAV capsid has rarely been observed in nonhuman primates (NHPs),[55] whereas some AAV serotypes do not induce immune responses in mice even though rodents generally have higher titers of pre-existing AAV antibodies.[54] Further, the most common animal models, including NHPs, do not host human commensal bacteria like *S. pyogenes* from which some gene editing components are derived and thus may not develop the same pre-existing immune responses expected in humans.[56]

Use of new technologies to predict immune responses to GTMPs is urgently required. New approaches include AI,[57,58] development of new animal models and approaches to humanizing animals, organoids, microphysiological (microfluidic) systems, and organ-on-chip approaches.[59–61] Current applications of organoids to gene therapy have not normally addressed immune responses.[60] Most of these approaches are still experimental and lack widespread regulatory acceptance, so future efforts should focus on providing guidance for validation and technical support. Access to innovation-type scientific and regulatory advice for sponsors developing such technologies is endorsed especially as many such technologies arise from academia where regulatory experience is often limited. To harmonize and accelerate GTMP development, continued regulatory guidance and advice are needed to support the development of alternative approaches for assessing immunogenicity-related risks, without relying on animal use. In this sense, FDA proposed in April 2025 a Roadmap to Reducing Animal Testing in Preclinical Safety Studies.

NONCLINICAL AND CLINICAL CROSS-CUTTING ASPECTS

Pre-existing humoral immunity

Prior exposure to viruses as part of natural infection results in development of pre-existing immunity. This is especially true for adenoviruses, AAV, and herpes simplex virus (HSV). Furthermore, some individuals may have previously received vaccination using viral vectors (either as approved products but also experimentally in clinical trials), especially adenoviruses, for example, as part of the COVID-19 vaccination campaign.[62,63] In general, patients with pre-existing immunity are excluded from clinical trials.[6,18,64] This decreases the treatable target population, potentially posing a threat to some AAV-based therapy business cases when the patient population is already very small, e.g., rare diseases.

Presence of antibodies is the most widely reported parameter of pre-existing immunity. For AAV, pre-existing seropositivity varies between approximately 30% to ≤60% or even 95% depending on the serotype, patient age, geography, and ethnicity.[64–72] Cross-reactivity between AAV serotypes has also been reported to be as high as 50% or more, due to general morphological conservation of the capsid protein,[73] with a total anti-AAV antibody prevalence ranging between 38% and 70%.[8,67] Similarly to AAV, HSV seroprevalence may also be as high as 90% in some populations.[74] Adenoviruses also show considerable seroprevalence in the general population although certain clades show reduced pre-existing immunity.[75] New, often animal-derived, adenovirus serotypes are being selected for therapeutic purposes based on low seroprevalence.[63,76] Finally, pre-existing humoral immunity to gene editing components such as CRISPR-Cas9 enzymes has also been reported in anywhere from 5% to 95% of the population, depending on the study and assay.[11]

Current guidelines are not specific about the requirements for performing screening for pre-existing immunity before conducting nonclinical studies (Table S1). nAbs against most AAV serotypes have been reported in all common animal models and vary between species and serotypes.[77–80] However, as the immune status of the animal model should mimic the clinical situation as closely as possible, pre-treatment of the animals with the vector to mimic the effects of pre-existing immunity may be considered, although this is not always practical. In addition, due to shortages of NHP, removing animals from studies due to high pre-existing AAV antibody titers adds cost, may not be feasible, and may not always be meaningful as the animal selection strategy will determine the patient selection strategy in clinical trials.

As the likelihood of exposure to a virus increases with age, prevalence of pre-existing immunity also increases. Seropositivity may thus potentially change between recruitment and treatment for example.[64,81] In the case of newborns up to approximately 1 year of age, maternal antibodies resulting from passive transfer may be present, and this may be difficult to model in animals.[64,66,68,72]

Regulatory focus on immunogenicity assessments for gene therapy vectors typically centers on standard gene replacement approaches, primarily using AAV, where an immune response is unwanted. However, as GTMPs also include therapeutic vaccines and oncolytic vectors, special immunogenicity considerations apply to these products. For example, AAV has been proposed as a vector for vaccine development,[63,64] raising concerns that widespread vaccination could affect seroprevalence and limit the use of certain AAV serotypes in future gene

therapies. The long-term benefits, risks, and ethical implications of AAV as a vaccine vector warrant careful consideration. On the other hand, for some oncolytic viruses, some pre-existing immunity may also improve functionality, and in these cases immunogenicity would be assessed as part of mechanistic studies.[82,83]

Pre-existing cellular immunity

The prevalence in the general population of pre-existing T cell immunity against gene therapy vectors is generally less studied than pre-existing humoral immunity. This is despite the fact that pre-existing cellular immunity could form during a wild-type AAV infection in early childhood and persist throughout life as a pool of memory T cells.[8,55] Capsid-specific T cells normally display high cross-reactivity[8,84,85] and show a memory phenotype[84,86,87] but are not frequently detected in the peripheral blood. There are several reasons for this, including the shorter persistence of effector T cells and low levels of central and memory T cells, which may respond with different kinetics to typical assay conditions.[88] Pre-existing reactive T cells to AAV are generally detected at a lower frequency in pediatric individuals compared to adults, presumably due to the reduced exposure to wild-type viruses in this population.[8] On the other hand, pre-existing T cell immunity to gene editing components has been reported in about 50%–100% of studied populations.[11] Overall, clinical associations between pre-existing humoral and cellular immunity and outcome are not always clear.

TREATMENT-INDUCED IMMUNITY

Innate immunity

The immediate immune response to gene therapy administration involves the innate immune response.[26,27] For example, fever, cytokine release, and elevated alanine transaminase (ALT) levels early (within 24 h) after AAV administration have been suggested to be the result, in part, of innate immune activation.[27,89] The innate immune response and its mediators and signaling pathways are not reviewed in detail here. Cells of the innate immune system include dendritic cells, monocyte/macrophages, and natural killer cells. The innate immune system also involves the role of various families of PRRs, which recognize pathogen-associated molecular patterns.[25,27,90] For example, unmethylated CpG dinucleotide-containing DNA is recognized by TLR9 during viral entry and uncoating. TLR9 signaling causes release of pro-inflammatory cytokines, chemokines, and a type I interferon response. These soluble effectors may be used as potential analytes for assessing innate immune system activation. Viral transduction may also activate the innate immune response via endoplasmic reticulum stress that results in an unfolded protein response.[9] Emerging data also suggest that the innate immune system may be involved in longer-term immunogenicity toward gene therapy products, for example, detection of double-stranded (ds)RNA and/or dsDNA as a result of ITR activity or other similar events during episomal maintenance.[27,91]

Synthetic mRNA including short guide RNA used in gene editing products has also been shown to trigger an innate cellular immune response leading to the upregulation of cytokines such as interferon-γ.[92] However, in therapeutic settings, mRNA is typically delivered encapsulated in lipid nanoparticles, which themselves are inherently immunostimulatory, as stated earlier, and, consequently, the relative contributions of each can be difficult to disentangle.[28,29] Innate immune detection of mRNA can be mitigated by incorporating modified bases such as pseudouridine in place of uridine or by using additional modifications such as 2′-*O*-methyl 3′-phosphorothioate.

Part of the innate immune response also includes complement activation.[26,27,93] Complement components have been shown to interact either directly with the AAV capsid or with immunocomplexes formed by anti-AAV antibodies and the capsid.[8,94] Activation of the complement system, especially after high dose gene therapy, has been linked to rare and serious adverse events including acute kidney injury as a result of atypical hemolytic uremic syndrome, a type of thrombotic microangiopathy.[93,94] Regulatory guidelines refer to possible requirements for complement assessment in preclinical studies (e.g., EMA/CAT/80183/2014; Table S1), but there are no clear clinical recommendations. Current requirements about complement assessments are thus unclear, particularly given the complexity of the complement system, which involves various soluble and cell surface-bound proteins that may play different roles depending on the disease context. Due to the sensitivity and risk of spontaneous complement activation in samples, reliable bioanalytical assessment requires careful planning regarding blood collection, handling, and storage. This may necessitate dedicated subgroups in nonclinical studies to ensure accurate assessment.[95,96] Nonetheless, preclinical models do not always predict human complement activation. In addition, there are various assay types for assessing complement activation or the role of individual components, which hinders standardization and comparison.

Adaptive immunity—Vector

The adaptive immune response to gene therapy is not summarized in detail and has been discussed extensively elsewhere.[6,8,9,97] Briefly, pre-existing or *de novo* humoral immunity to a gene therapy vector results in antibody binding and potential neutralization and inhibition of transduction. Vector particles can also be processed in transduced cells and presented by major histocompatibility class I to evoke a $CD8^+$ cytotoxic T lymphocyte (CTL) response that eliminates transduced cells. For example, after liver gene therapy, cytolysis of transduced cells detected as the release of liver enzymes, such as ALT, usually starts 1–4 weeks post-AAV injection.[8,89,98] Indeed, high-dose AAV gene therapy has been associated with acute liver immunotoxicity that has resulted in the deaths of several patients with spinal muscular atrophy (SMA) and Duchenne muscular dystrophy (DMD).[89,99] A capsid-specific CTL response by infiltrating $CD8^+$ T cells was considered the likely cause in patients with SMA.[99]

Vector immunogenicity is dependent on many factors, and these include the vector dose, the target organ, the route of administration, and the structural and biochemical characteristics of the vector.[6,8,21,25] For example, compared to AAV, adenoviruses are more efficient in activating $CD8^+$ T cells resulting in robust CTL and Th1 responses.[100,101] This observation has been linked to different innate immune detection of adenoviruses as well as better transduction of professional APCs. For these reasons, adenoviruses are generally considered more appropriate as vaccines and immunomodulatory or oncolytic products

rather than vehicles for long-term therapeutic transgene expression.[101] Anti-AAV capsid cellular immune responses are generally detected from as early as 14 days following dose administration and may often be sporadic as well as dose and disease dependent, potentially declining over 1 year.[55,89]

Adaptive immunity—Transgene product

Immune responses to the transgene product are less frequently reported than immunogenicity to the vector, but there are some reports, especially in skeletal muscle where the underlying presence of inflammation due to disease pathophysiology is hypothesized to play a role.[8,20,98,100,102–104] There are also several confounding factors that make interpretation of anti-transgene immunogenicity more difficult. Many diseases have variable genetic causes. Mutations may result in a complete absence of endogenous protein (e.g., null or cross-reactive immunological material [CRIM]-negative patients in Pompe's disease) or imply the presence of varying quantities of residual protein perhaps with qualitatively decreased functionality (e.g., CRIM-positive).[105] Other cases where anti-transgene immunity may be important include expressing a protein in an alternative tissue location or when vaccinating against cancer using neo-antigens.[16] Immunogenicity may also depend on the subcellular localization and whether or not it is secreted or membrane bound. Transgene products may also be modified compared to the endogenous protein, increasing immunogenicity. In some cases, administration of an exogenous protein akin to the transgene product may be the standard of care. Examples of this include enzyme replacement therapy (ERT) or administration of clotting factors, which are known to give rise to immunogenicity and inhibitors.[106,107] Clinical trial populations are generally selected to avoid those with pre-existing humoral immunity (inhibitors). Furthermore, the risks associated with a GTMP inducing an immune response that may reduce the patient's response to other therapies such as ERT is an important consideration in the case that clinical benefit from the GTMP is not achieved.

Tissue context plays a major role in shaping immune outcomes; for example, skeletal muscle may be potentially more immunogenic than other sites of delivery.[102] In this case, CTLs directed against various transgene products have been observed following gene therapy for DMD or other conditions targeting skeletal muscle.[9,103] In relation to chimeric antigen receptor (CAR) T cells, most approved products, except for ciltacabtagene autoleucel, utilize a murine portion of the CAR (murine single chain variable fragment; scFv), which may be immunogenic and activate innate and adaptive responses although the clinical significance is not clear.[108]

Tolerance

The concept of immunological tolerance has several potential impacts on gene therapy. On one hand, it is well established that liver-directed gene therapy can induce systemic tolerance toward the transgene and immunosuppressive T_{regs} play an important role in this.[109–111] The immunosuppressive response is induced by presentation of transgene-derived epitopes by liver resident APCs (e.g., Kupffer cells) and subsequent induction of a transgene-specific T_{reg} response. T_{regs} suppress the humoral and cellular immune responses toward the transgene by multiple mechanisms including release of anti-inflammatory cytokines such as interleukin (IL)-10 and transforming growth factor β.[109] Strategies to induce tolerance to GTMPs include prophylactic oral administration of the immunogenic protein before AAV administration.[112] On the other hand, in the case of cancer gene therapy, breaking tolerance may be necessary to assure an adequate immune response against self-tumor antigens although neo-antigens may already induce immune responses.[16]

Autoimmunity remains largely a theoretical concern for some GTMPs although reports of autoimmunity following gene therapy in the clinical setting are extremely rare. Several regulatory guidelines highlight the potential for cross-reactive or bystander autoimmune responses, especially upon prolonged or repeat exposure, and the need to assess these responses as part of GTMP development (Table S1). Anti-nuclear antibodies (ANAs) are also mentioned as a possible autoimmune analytes; however, ANAs may also be present in up to 30%–40% of the healthy population.[113] In terms of cancer gene therapy, one of the principal concerns comes from the use of self-tumor antigens or neo-antigens to drive immune responses[114,115,116,117]. It should be noted that a recent meta-analysis including data from 55 human clinical trials, abstracts, case reports, and unpublished data representing 3,323 patients treated with whole-tissue autologous therapeutic vaccines for various cancers showed no risk of autoimmunity.[116] Autoimmunity may also develop in the context of immunomodulation such as concomitant use of immune checkpoint inhibitors.[117]

Immunomodulation

For most GTMP applications, immunomodulation regimens usually refer only to application of immunosuppression, which is now essentially always adopted clinically, including prophylactically, for improving safety and for sustaining efficacy.[5,18,118] For example, immunosuppressive regimens are also used in ocular and CNS clinical trials despite the supposed immune-privileged status of these tissues.[119,120]

The most common immunosuppressants administered clinically are corticosteroids, sirolimus, ciclosporin, tacrolimus, and mycophenolate mofetil.[5] Each agent has its own limitations and technical considerations such as use preference, dose level and duration of treatment, tapering requirements, specific safety and adverse event profiles, and suitability for pediatric use. Particular safety concerns from long-term immunosuppression include increased susceptibility to infection and the possible development of malignancy. Some agents such as corticosteroids may also have a positive effect on efficacy outcomes in some circumstances such as in DMD where it is generally part of the standard of care.[121]

Other agents used in immunosuppressive protocols include rituximab, an anti-CD20 monoclonal antibody, and imlifidase, an immunoglobulin G -degrading enzyme. These substances target B cell-specific molecules and are used to lower pre-existing anti-AAV antibodies, enhancing transduction rates and transgene expression.[18] Proteasome inhibitors like bortezomib and carfilzomib improve AAV efficacy by preventing degradation of ubiquitinated AAV particles, while chemotherapeutics such as teniposide act as transduction enhancers by promoting

conversion of AAV genome into dsDNA or inhibiting DNA damage response proteins that would hamper that process.[122,123] Another novel approach to immunosuppression is ibrutinib, which disrupts B cell receptor and cytokine receptor pathways including downregulation of PD-L1 pathways and reducing IL-10 production.[124] Co-administration of ibrutinib with rapamycin, a potent and selective mTOR inhibitor, markedly suppressed primary antibody generation, attenuated recall responses, and reduced the number of antibody-secreting plasma cells following AAV gene therapy in mice; however, this regimen did not eliminate memory B cell formation, nor did it restrain pre-existing antibodies to exert their inhibitory effect on transduction.[124,125] One method to tackle pre-existing anti-AAV antibodies is frequent sessions of plasmapheresis following AAV gene therapy allowing re-dosing.[126,127] Removal of AAV antibodies by plasmapheresis in AAV seropositive animals resulted in high-level transduction, which was comparable with that of AAV seronegative animals.[127]

Gene-modified hematopoietic stem cell and CAR T therapies also often use prior myeloablation/lymphoablation to optimize marrow repopulation.[92,128] Patients who receive such conditioning prior to receiving such therapies usually have better responses. Despite this, conditioning has many limitations including induction of a transient immunosuppression, loss of immune memory (making patients susceptible to opportunistic infections or viral reactivation), and age-associated differences in conditioning response.[92,128] A typical conditioning regimen involves use of chemotherapy, such as a combination of fludarabine and cyclophosphamide, although other regimens including use of radiation and novel approaches are being developed. A key aspect to selection of conditioning regimen can also be the type of cell to be transplanted and the underlying indication, since some gene therapy-corrected cells may have survival advantages meaning that less intense regimens may be applied. The development of conditioning and the associated immune consequences are further in the spotlight as more clinical experience is gained in the race to develop off-the-shelf allogeneic approaches. Related to the use of conditioning is also the fact that cancer patients may have received multiple treatments including cytotoxic and/or immunosuppressive therapies that may affect the immune system and the mechanistic response to the therapeutic vaccine or oncolytic virus.

Immunosuppression may be associated with persistent infection as a specific safety concern. In addition, immunosuppression may also affect shedding. This is especially in the case of oncolytic gene therapies, since the administered vector may be cleared differently depending on the activity status of the immune system, and additional monitoring of immunosuppressed patients is required.[129]

In contrast to immunosuppression for "classical" gene therapy approaches, the use of immune enhancement has gradually gained traction for some GTMP approaches to boost efficacy as part of product mechanism. Such approaches include use of adjuvants in therapeutic vaccines[16] or co-administration of immune checkpoint inhibitors (ICIs) in combination with cancer vaccines, oncolytic viruses, and CAR T cells.[130–132] Furthermore, some cancer vaccine or oncolytic approaches have also used pro-inflammatory molecules, such as IL-2 and IL-12, as part of their mechanism of action.[133,134] However, these approaches are often hampered by the animal models, which are limited by the type of tumor cell that can be implanted and how they recapitulate the human tumor microenvironment. These animal models also generally require humanization, are limited in terms of ICI activity or pharmacokinetics (especially for combinations), and show poor predictability of irAEs.[135–137]

The regulatory requirements for immunomodulation during nonclinical development of GTMPs are currently not clear (Table S1). In principle, pivotal toxicity and safety assessments should try to represent the clinical administration conditions as closely as possible, and this could include the use of a representative clinical immunomodulatory regimen. Immunomodulation generally changes according to dosing, timing, and route of administration.[5,6,8] However, as clinical immunomodulatory regimens may vary in duration and dose according to the response and monitoring of patients, this is hard to model in animals. In addition, the efficacy of some regimens or agents may not be fully established in all animal models or species. Further systematic studies or guidance on this would be beneficial. In addition, use of immunosuppression in animals may add additional cost to studies without necessarily adding predictive value.

Overall, more guidance on immunomodulation requirements including requirements for preclinical studies and the limitations and alternatives to preclinical models is needed. The risks associated with the immunomodulation should be considered as part of the overall product benefit:risk assessment. As immunomodulatory agents are used concomitantly with GTMPs (especially immediately around the time of GTMP administration), it is important to consider the implications of interactions and the difficulty of assigning causality to safety signals to a particular treatment.

BIOANALYTICAL METHODS

Bioanalytical methods to determine the consequences of immunogenicity or measure other biomarkers in nonclinical and/or clinical biological samples (matrices) need to be qualified or validated according to guidelines (Table S2). There are several recent references and initiatives that describe considerations for the different types of immunogenicity assays relevant to GTMPs.[55,71,138–142] Several key technical aspects for developing the assays are described in detail in these references and are not repeated here. General aspects include the need to show a qualification or that the assay is fit for purpose at early stages of development with full validation coming at later stages in time for pivotal studies. Sample and analyte stability is also a potential issue. In many cases, it may be acceptable to obtain and store biological samples for future analysis should the need arise and/or when a suitable method is available. However, the time of storage of these samples prior to analysis needs to be covered by stability as part of the method qualification/validation. This stability and sample storage may imply additional cost or sample loss. As many assays may show intermittent or fluctuating results, it is generally recommended that at least two pre-treatment samples be collected to assure that an adequate baseline can be established.[55]

There are several broader regulatory issues regarding development of bioanalytical methods for GTMPs. Firstly, methods

and reagents (including vector reference standards) are not standardized.[65,71] For example, developers have adopted diverse approaches for bioanalytical method development and validation while applying varying approaches to assay cutoff determination in the absence of specific GTMP guidelines. Each method is usually fit to purpose and comparison between studies is difficult.[143,144] Secondly, assays may also be fit for purpose at early stages of development, but, in the case of rare diseases, these data may go on to support claims of efficacy and safety, and thus the importance of the assay validation may come into play. Approaches to evolving assays during development are thus welcome. Compounding this issue is often the limited reporting of key assay parameters and functionality in published papers, which makes systematic comparisons difficult.[5,145] Thirdly, as many gene therapy developments arise from academia, a clearer understanding of bioanalytical method requirements even at this level of development may be beneficial. It could improve method reporting requirements as well as later technology transfer while generating more reliable early-stage data to support scientific advice and justification for reducing study requirements and thus developmental costs. Improving the capacity to broadly analyze and compare different gene therapy approaches and bioanalytical methods is one potential way to accelerate GTMP development. There are ongoing initiatives to address this issue including from ARDAT.

Although monitoring of pre-existing or treatment-induced T cell immunity is not as widely described in guidelines as humoral immunity, the importance of the cellular immune response is reflected in guidelines from both EMA and FDA (Table S1). Guidelines describe the importance of investigating and controlling cellular immunity, both pre-existing and treatment induced, in relation to timing of treatment and possible re-dosing, but without specific assay recommendations. The FDA hemophilia guideline is more specific as it recommends to do short-term monitoring of the T cell immune response in peripheral blood mononuclear cells (PBMCs) by ELISpot assays in addition to the vector-related antibodies and to increase testing frequency if immune-mediated hepatic dysfunction is suspected.

Companion diagnostics

Within drug development, the recent implementation of medical device and *in vitro* diagnostic (IVD) legislation is having an important impact on the development of GTMPs, including implementation of the new European *In Vitro* Diagnostic Medical Devices Regulation (IVDR) 2017/746/EU. In particular, the requirements for developing a CDx are becoming increasingly critical.[64,71,139] In Europe, a CDx is defined as an IVD test that supports the safe and effective use of a specific corresponding medicinal product, for example, by identifying patients that are suitable or unsuitable for treatment such as those with critical levels of pre-existing immunity (Regulation 2017/746/EU).

Several commercially approved, systemically administered AAV products have co-developed a CDx in the EU and USA. AAV5 DetectCDx is a CDx used in the selection of patients eligible for treatment with valoctocogene roxaparvovec-rvox (Roctavian), a gene therapy product indicated for treatment of severe hemophilia A. The same CDx previously received a Conformité Européenne (CE) mark as a medical device in 2020 under the previous *In Vitro* Diagnostic Medical Devices Directive and more recently in late 2023 obtained a new CE mark under the IVDR. The final approved indication for valoctocogene roxaparvovec-rvox includes the restriction limiting use to patients "without detectable antibodies to AAV5." In addition, the nAb-Cyte Anti-AAVRh74var HB-FE Assay CDx was also similarly developed as an FDA post-marketing requirement for Pfizer's hemophilia B gene therapy Durveqtix/Beqvez (fidanacogene elaparvovec-dzkt).

There are currently several regulatory issues regarding the co-development of a CDx. Some of these aspects include the need for the CDx to undergo a conformity assessment by a notified body (the European competent authority responsible for approval of medical devices, which differs from the competent authorities who will approve ATMPs). In the USA, approval of a CDx or ATMP is made by the FDA. Timing is also essential since there is a need to validate the assay before a clinically relevant cutoff can be established, which requires clinical studies. In addition, due to inherent methodological differences, it is effectively meaningless to make comparisons of cutoffs and titers across assays.

PEDIATRIC REGULATORY ISSUES

Many of the indications targeted by gene therapies are for pediatric conditions, and there are several important and specific regulatory and scientific considerations for understanding and assessing immunity in this special population.

It is known that the use of corticosteroids is associated with significant morbidity. The adverse events that are associated with long-term systemic (oral or parenteral) use of corticosteroids in children include growth suppression, decreased bone mineralization, osteoporosis, fractures and aseptic bone necrosis, suppression of the hypothalamic-pituitary-adrenal axis, adrenal gland atrophy, Cushingoid appearance, hyperglycaemia and diabetes, cardiovascular disease, dyslipidaemia, dermatological events (acne and red striae), gastrointestinal events, psychiatric and cognitive disturbances, and increased risk for infection.[146] Adrenal gland atrophy may result in life-threatening adrenal insufficiency associated with illness, injury, or surgery, with a requirement for prophylactic doses of corticosteroids as part of treatment regimen during these periods.[146] Many immunomodulator products are also not authorized for pediatric use meaning there may be some concerns around dosing, off-label use, and/or developmental toxicity.

A child exposed to gene therapy at a very early age may develop significant cross-reactivity to different serotypes of the same vector, which may limit re-administration or future gene therapy treatment options of the same modality.[68,72] This is an important consideration since some transgene expressions, and thus efficacy, may be naturally lost over time due to the non-integrating nature of the vector and natural pediatric development.

In infants, particularly neonates, anti-vector antibodies may be detected as a result of maternal transfer via the placenta or from ingestion of breast milk although this will decline with time and increase again due to natural infection and seroconversion.[68,72,147] The limited volume of blood draws that can be

made in young participants is also a limiting factor for immunogenicity assessments, where high blood volumes may be required for all appropriate testing.

Pediatric individuals also require periodic vaccination as part of routine health care. The possible interference of vaccines with gene therapy products and/or the immunomodulatory requirements is a particular concern. In addition, there may be many local or regional differences in vaccination schedules that should be considered in relation to clinical trial design. Many vaccines will also be contraindicated in patients receiving immunosuppression, especially live attenuated vaccines.

CONCLUSIONS AND REGULATORY RECOMMENDATIONS

The aim of the current review article was to provide an updated assessment of the international regulatory requirements for assessment of immunogenicity and immunomodulation relevant for global development and licensing of GTMPs. The current guidelines have been identified, and key aspects qualified and discussed. Based on our assessment, we make the following general conclusions and proposals for future guidance on this topic.

(1) A stand-alone guidance on immunogenicity and immunomodulation requirements for GTMPs does not currently exist and would be welcome.
(2) While it is recognized that GTMPs are diverse and immune responses vary according to many factors such as product design manufacturing, route of administration, and dose, a guideline that covers the various technologies and mechanistic scenarios would be welcome (i.e., one size does not fit all). A broad approach may also help identify future areas for investigation.
(3) Regulatory guidance (especially in the EU) clarifying the classification and requirements of therapeutic vaccines for treatment of cancer and chronic infectious diseases would be welcome.
(4) Recommendations and clarifications regarding preclinical requirements for assessing pre-existing immunity and immunomodulation protocols in animal models would be welcome.
(5) Autoimmunity is considered a potential risk, but currently recommendations and data expectations regarding autoimmunity assessments are limited.
(6) Pediatric guidance and considerations regarding clinical assessment requirements and immunosuppression options would be welcome.
(7) Increased guidance on pharmacovigilance requirements for safety reporting of irAEs would be well received, especially given that concomitant immunomodulation may complicate interpretation of the irAE.
(8) Approaches for developing and implementing risk management strategies including any post-marketing requirements for LTFU of irAEs.
(9) More regulatory support and continued access to advice procedures for development of a CDx and bioanalytical methods would be positive.
(10) Guidance and forums from regulators on how to develop novel testing methodologies for assessing immunogenicity that would reduce animal usage requirements would be welcome.
(11) Continued regulatory support, education, and access to advice procedures for early-stage (academic) development to facilitate supportive (non-pivotal) immunogenicity data generation. This would support early risk assessments and later animal reduction as well as technology transfer of related assay methods.
(12) Journal articles reporting on immunogenicity-related bioanalytical methods should include a minimum of information such as assay method used, sensitivity, minimum required dilution, positive control antibody used, and titer determination.
(13) Journal articles reporting on immunomodulatory regimens should include a clearer description of the regimen used including name of the agent, dose level, duration, and treatment schedule (such as tapering).
(14) In terms of regulatory documentation, increased clarity on the expected localization and evaluation of immunogenicity and immunomodulatory data in a regulatory dossier would be appreciated.

ACKNOWLEDGMENTS

This project has received funding from the Innovative Medicines Initiative 2 Joint Undertaking (JU) under grant agreement no. 945473. The JU receives support from the European Union's Horizon 2020 research and innovation program and the European Federation of Pharmaceutical Industries and Associations.

AUTHOR CONTRIBUTIONS

Conceptualization, C.J.M., T.P.H., and M.A.; writing – original draft, C.J.M., J. G., M.B., F.S., M.R.S., P.C., A.H., and T.L.; writing – review and editing, C.J.M., J.G., M.B., F.S., M.R.S., P.C., T.L., N.C., T.P.H., and M.A.

DECLARATION OF INTERESTS

P.C. is an employee and shareholder of Sanofi. Additional affiliation is Department of Pharmacy, University of Pisa, Pisa, Italy. M.A. is co-founder and shareholder of Blackfin Bio and Crucible Therapeutics. N.C. is an employee and shareholder of Asphalion.

SUPPLEMENTAL INFORMATION

Supplemental information can be found online at https://doi.org/10.1016/j.xcrm.2025.102422.

REFERENCES

1. Wilkins, G.C., Lanyi, K., Inskip, A., Ogunbayo, O.J., Brhlikova, P., and Craig, D. (2023). A pipeline analysis of advanced therapy medicinal products. Drug Discov. Today *28*, 103549.
2. Kassir, Z., Sarpatwari, A., Kocak, B., Kuza, C.C., and Gellad, W.F. (2020). Sponsorship and Funding for Gene Therapy Trials in the United States. JAMA *323*, 890–891.
3. Olesti, E., Nuevo, Y., Bachiller, M., Guillen, E., Bascuas, J., Varea, S., Saez-Peñataro, J., and Calvo, G. (2024). Academic challenges on advanced therapy medicinal products' development: a regulatory perspective. Cytotherapy *26*, 221–230.

CellPress
OPEN ACCESS

4. Olry de Labry-Lima, A., Ponce-Polo, A., García-Mochón, L., Ortega-Ortega, M., Pérez-Troncoso, D., and Epstein, D. (2023). Challenges for Economic Evaluations of Advanced Therapy Medicinal Products: A Systematic Review. Value Health *26*, 138–150.
5. Vrellaku, B., Sethw Hassan, I., Howitt, R., Webster, C.P., Harriss, E., McBlane, F., Betts, C., Schettini, J., Lion, M., Mindur, J.E., et al. (2024). A systematic review of immunosuppressive protocols used in AAV gene therapy for monogenic disorders. Mol. Ther. *32*, 3220–3259.
6. Mingozzi, F., and High, K.A. (2017). Overcoming the Host Immune Response to Adeno-Associated Virus Gene Delivery Vectors: The Race Between Clearance, Tolerance, Neutralization, and Escape. Annu. Rev. Virol. *4*, 511–534.
7. Shirley, J.L., de Jong, Y.P., Terhorst, C., and Herzog, R.W. (2020). Immune Responses to Viral Gene Therapy Vectors. Mol. Ther. *28*, 709–722.
8. Costa Verdera, H., Kuranda, K., and Mingozzi, F. (2020). AAV Vector Immunogenicity in Humans: A Long Journey to Successful Gene Transfer. Mol. Ther. *28*, 723–746.
9. Ertl, H.C.J. (2022). Immunogenicity and toxicity of AAV gene therapy. Front. Immunol. *13*, 975803.
10. Drysdale, C.M., Tisdale, J.F., and Uchida, N. (2020). Immunoresponse to Gene-Modified Hematopoietic Stem Cells. Mol. Ther. Methods Clin. Dev. *16*, 42–49.
11. Ewaisha, R., and Anderson, K.S. (2023). Immunogenicity of CRISPR therapeutics-Critical considerations for clinical translation. Front. Bioeng. Biotechnol. *11*, 1138596.
12. Wagner, D.L., Fritsche, E., Pulsipher, M.A., Ahmed, N., Hamieh, M., Hegde, M., Ruella, M., Savoldo, B., Shah, N.N., Turtle, C.J., et al. (2021). Immunogenicity of CAR T cells in cancer therapy. Nat. Rev. Clin. Oncol. *18*, 379–393.
13. Baker, A.H., and Herzog, R.W. (2020). Did Dendritic Cell Activation, Induced by Adenovirus-Antibody Complexes, Play a Role in the Death of Jesse Gelsinger? Mol. Ther. *28*, 704–706.
14. Somanathan, S., Calcedo, R., and Wilson, J.M. (2020). Adenovirus-Antibody Complexes Contributed to Lethal Systemic Inflammation in a Gene Therapy Trial. Mol. Ther. *28*, 784–793.
15. Maina, A., and Foster, G.R. (2024). Hepatitis after gene therapy, what are the possible causes? J. Viral Hepat. *31*, 14–20.
16. Fan, T., Zhang, M., Yang, J., Zhu, Z., Cao, W., and Dong, C. (2023). Therapeutic cancer vaccines: advancements, challenges, and prospects. Signal Transduct. Target. Ther. *8*, 450.
17. Park, P.G., Fatima, M., An, T., Moon, Y.E., Woo, S., Youn, H., and Hong, K.J. (2024). Current development of therapeutic vaccines for the treatment of chronic infectious diseases. Clin. Exp. Vaccine Res. *13*, 21–27.
18. Chu, W.S., and Ng, J. (2021). Immunomodulation in Administration of rAAV: Preclinical and Clinical Adjuvant Pharmacotherapies. Front. Immunol. *12*, 658038.
19. Leborgne, C., Barbon, E., Alexander, J.M., Hanby, H., Delignat, S., Cohen, D.M., Collaud, F., Muraleetharan, S., Lupo, D., Silverberg, J., et al. (2020). IgG-cleaving endopeptidase enables in vivo gene therapy in the presence of anti-AAV neutralizing antibodies. Nat. Med. *26*, 1096–1101.
20. Samelson-Jones, B.J., Finn, J.D., Favaro, P., Wright, J.F., and Arruda, V. R. (2020). Timing of Intensive Immunosuppression Impacts Risk of Transgene Antibodies after AAV Gene Therapy in Nonhuman Primates. Mol. Ther. Methods Clin. Dev. *17*, 1129–1138.
21. Yang, T.Y., Braun, M., Lembke, W., McBlane, F., Kamerud, J., DeWall, S., Tarcsa, E., Fang, X., Hofer, L., Kavita, U., et al. (2022). Immunogenicity assessment of AAV-based gene therapies: An IQ consortium industry white paper. Mol. Ther. Methods Clin. Dev. *26*, 471–494.
22. Shankar, G., Arkin, S., Cocea, L., Devanarayan, V., Kirshner, S., Kromminga, A., Quarmby, V., Richards, S., Schneider, C.K., Subramanyam, M., et al. (2014). Assessment and reporting of the clinical immunogenicity of therapeutic proteins and peptides-harmonized terminology and tactical recommendations. AAPS J. *16*, 658–673.
23. Shankar, G., Devanarayan, V., Amaravadi, L., Barrett, Y.C., Bowsher, R., Finco-Kent, D., Fiscella, M., Gorovits, B., Kirschner, S., Moxness, M., et al. (2008). Recommendations for the validation of immunoassays used for detection of host antibodies against biotechnology products. J. Pharm. Biomed. Anal. *48*, 1267–1281.
24. Devanarayan, V., Smith, W.C., Brunelle, R.L., Seger, M.E., Krug, K., and Bowsher, R.R. (2017). Recommendations for Systematic Statistical Computation of Immunogenicity Cut Points. AAPS J. *19*, 1487–1498.
25. Wright, J.F. (2020). Codon Modification and PAMPs in Clinical AAV Vectors: The Tortoise or the Hare? Mol. Ther. *28*, 701–703.
26. Muhuri, M., Maeda, Y., Ma, H., Ram, S., Fitzgerald, K.A., Tai, P.W., and Gao, G. (2021). Overcoming innate immune barriers that impede AAV gene therapy vectors. J. Clin. Investig. *131*, e143780.
27. Wang, Y., and Shao, W. (2023). Innate Immune Response to Viral Vectors in Gene Therapy. Viruses *15*, 1801.
28. Lee, Y., Jeong, M., Park, J., Jung, H., and Lee, H. (2023). Immunogenicity of lipid nanoparticles and its impact on the efficacy of mRNA vaccines and therapeutics. Exp. Mol. Med. *55*, 2085–2096.
29. Chen, S.P., and Blakney, A.K. (2024). Immune response to the components of lipid nanoparticles for ribonucleic acid therapeutics. Curr. Opin. Biotechnol. *85*, 103049.
30. Remes, A., Basha, D.I., Puehler, T., Borowski, C., Hille, S., Kummer, L., Wagner, A.H., Hecker, M., Soethoff, J., Lutter, G., et al. (2021). Alginate hydrogel polymers enable efficient delivery of a vascular-targeted AAV vector into aortic tissue. Mol. Ther. Methods Clin. Dev. *21*, 83–93.
31. Bashor, C.J., Hilton, I.B., Bandukwala, H., Smith, D.M., and Veiseh, O. (2022). Engineering the next generation of cell-based therapeutics. Nat. Rev. Drug Discov. *21*, 655–675.
32. Faust, S.M., Bell, P., Cutler, B.J., Ashley, S.N., Zhu, Y., Rabinowitz, J.E., and Wilson, J.M. (2013). CpG-depleted adeno-associated virus vectors evade immune detection. J. Clin. Investig. *123*, 2994–3001.
33. Khalifeh, M., and Salman, H. (2025). Engineering resilient CAR T cells for immunosuppressive environment. Mol. Ther. *33*, 2391–2405.
34. Tan, F., Dong, Y., Qi, J., Yu, W., and Chai, R. (2025). Artificial Intelligence-Based Approaches for AAV Vector Engineering. Adv. Sci. *12*, e2411062.
35. Srivastava, A., Mallela, K.M.G., Deorkar, N., and Brophy, G. (2021). Manufacturing Challenges and Rational Formulation Development for AAV Viral Vectors. J. Pharm. Sci. *110*, 2609–2624.
36. Wright, J.F. (2014). Product-Related Impurities in Clinical-Grade Recombinant AAV Vectors: Characterization and Risk Assessment. Biomedicines *2*, 80–97.
37. Yamaguchi, Y., Ishii, K., Koizumi, S., Sakaue, H., Maruno, T., Fukuhara, M., Shibuya, R., Tsunaka, Y., Matsushita, A., Bandoh, K., et al. (2024). Glycosylation of recombinant adeno-associated virus serotype 6. Mol. Ther. Methods Clin. Dev. *32*, 101256.
38. Bing, S.J., Justesen, S., Wu, W.W., Sajib, A.M., Warrington, S., Baer, A., Thorgrimsen, S., Shen, R.F., and Mazor, R. (2022). Differential T cell immune responses to deamidated adeno-associated virus vector. Mol. Ther. Methods Clin. Dev. *24*, 255–267.
39. Bing, S.J., Warrington, S., and Mazor, R. (2023). Low cross reactivity between wild type and deamidated AAV can lead to false negative results in immune monitoring T-cell assays. Front. Immunol. *14*, 1211529.
40. Rumachik, N.G., Malaker, S.A., Poweleit, N., Maynard, L.H., Adams, C. M., Leib, R.D., Cirolia, G., Thomas, D., Stamnes, S., Holt, K., et al. (2020). Methods Matter: Standard Production Platforms for Recombinant AAV Produce Chemically and Functionally Distinct Vectors. Mol. Ther. Methods Clin. Dev. *18*, 98–118.
41. Margine, I., Martinez-Gil, L., Chou, Y.Y., and Krammer, F. (2012). Residual baculovirus in insect cell-derived influenza virus-like particle preparations enhances immunogenicity. PLoS One *7*, e51559.
42. Mora, J., Forman, D., Hu, J., Ijantkar, A., Gokemeijer, J., Kolaja, K.L., Picarillo, C., Jawa, V., Yue, H., Lamy, J., et al. (2024). Immunogenicity

Risk Assessment of Process-Related Impurities in An Engineered T Cell Receptor Cellular Product. J. Pharm. Sci. *113*, 2151–2160.

43. Silva, A.K.A., Morille, M., Piffoux, M., Arumugam, S., Mauduit, P., Larghero, J., Bianchi, A., Aubertin, K., Blanc-Brude, O., Noël, D., et al. (2021). Development of extracellular vesicle-based medicinal products: A position paper of the group "Extracellular Vesicle translatiOn to clinicaL perspectiVEs - EVOLVE France". Adv. Drug Deliv. Rev. *179*, 114001.

44. Xia, Y., Zhang, J., Liu, G., and Wolfram, J. (2024). Immunogenicity of Extracellular Vesicles. Adv. Mater. *36*, e2403199.

45. Zhu, X., Badawi, M., Pomeroy, S., Sutaria, D.S., Xie, Z., Baek, A., Jiang, J., Elgamal, O.A., Mo, X., Perle, K.L., et al. (2017). Comprehensive toxicity and immunogenicity studies reveal minimal effects in mice following sustained dosing of extracellular vesicles derived from HEK293T cells. J. Extracell. Vesicles *6*, 1324730.

46. Buzas, E.I. (2023). The roles of extracellular vesicles in the immune system. Nat. Rev. Immunol. *23*, 236–250.

47. Jawa, V., Joubert, M.K., Zhang, Q., Deshpande, M., Hapuarachchi, S., Hall, M.P., and Flynn, G.C. (2016). Evaluating Immunogenicity Risk Due to Host Cell Protein Impurities in Antibody-Based Biotherapeutics. AAPS J. *18*, 1439–1452.

48. Brimble, M.A., Cheng, P.H., Winston, S.M., Reeves, I.L., Souquette, A., Spence, Y., Zhou, J., Wang, Y.D., Morton, C.L., Valentine, M., et al. (2022). Preventing packaging of translatable P5-associated DNA contaminants in recombinant AAV vector preps. Mol. Ther. Methods Clin. Dev. *24*, 280–291.

49. FDA Center for Biologics Evaluation and Research (CBER). Guidance for Industry: Chemistry, Manufacturing, and Control (CMC) Information for Human Gene Therapy Investigational New Drug Applications (INDs) Maryland, USA: Food and Drug Administration (FDA); 2020 [updated January 2020. Available from: https://www.fda.gov/media/113760/download.

50. Pham, N.B., and Meng, W.S. (2020). Protein aggregation and immunogenicity of biotherapeutics. Int. J. Pharm. *585*, 119523.

51. Canals, J.M., Romania, P., Belio-Mairal, P., Nic, M., Dibusz, K., Novotny, T., Busquet, F., Rossi, F., Straccia, M., and Daskalopoulos, E.P. (2022). Advanced Non-animal Models in Biomedical Research: Immunogenicity Testing for Advanced Therapy Medicinal Products (JRC126997) (Publications Office of the European Union). https://publications.jrc.ec.europa.eu/repository/bitstream/JRC126997/JRC126997_01.pdf.

52. Martino, A.T., and Markusic, D.M. (2020). Immune Response Mechanisms against AAV Vectors in Animal Models. Mol. Ther. Methods Clin. Dev. *17*, 198–208.

53. Diehl, R., Ferrara, F., Müller, C., Dreyer, A.Y., McLeod, D.D., Fricke, S., and Boltze, J. (2017). Immunosuppression for in vivo research: state-of-the-art protocols and experimental approaches. Cell. Mol. Immunol. *14*, 146–179.

54. Ertl, H.C.J. (2019). Preclinical models to assess the immunogenicity of AAV vectors. Cell. Immunol. *342*, 103722.

55. Gorovits, B., Azadeh, M., Buchlis, G., Fiscella, M., Harrison, T., Havert, M., Janetzki, S., Jawa, V., Long, B., Mahnke, Y.D., et al. (2023). Evaluation of Cellular Immune Response to Adeno-Associated Virus-Based Gene Therapy. AAPS J. *25*, 47.

56. Wagner, D.L., Peter, L., and Schmueck-Henneresse, M. (2021). Cas9-directed immune tolerance in humans-a model to evaluate regulatory T cells in gene therapy? Gene Ther. *28*, 549–559.

57. Wec, A.Z., Lin, K.S., Kwasnieski, J.C., Sinai, S., Gerold, J., and Kelsic, E. D. (2021). Overcoming Immunological Challenges Limiting Capsid-Mediated Gene Therapy With Machine Learning. Front. Immunol. *12*, 674021.

58. Farzan, R. (2024). Artificial intelligence in Immuno-genetics. Bioinformation *20*, 29–35.

59. Marx, U., Akabane, T., Andersson, T.B., Baker, E., Beilmann, M., Beken, S., Brendler-Schwaab, S., Cirit, M., David, R., Dehne, E.M., et al. (2020). Biology-inspired microphysiological systems to advance patient benefit and animal welfare in drug development. ALTEX *37*, 365–394.

60. Ramamurthy, R.M., Atala, A., Porada, C.D., and Almeida-Porada, G. (2022). Organoids and microphysiological systems: Promising models for accelerating AAV gene therapy studies. Front. Immunol. *13*, 1011143.

61. Wagar, L.E., Salahudeen, A., Constantz, C.M., Wendel, B.S., Lyons, M. M., Mallajosyula, V., Jatt, L.P., Adamska, J.Z., Blum, L.K., Gupta, N., et al. (2021). Modeling human adaptive immune responses with tonsil organoids. Nat. Med. *27*, 125–135.

62. Chavda, V.P., Bezbaruah, R., Valu, D., Patel, B., Kumar, A., Prasad, S., Kakoti, B.B., Kaushik, A., and Jesawadawala, M. (2023). Adenoviral Vector-Based Vaccine Platform for COVID-19: Current Status. Vaccines (Basel) *11*, 432.

63. Aledo-Serrano, A., Gil-Nagel, A., Isla, J., Mingorance, A., Mendez-Hermida, F., and Hernandez-Alcoceba, R. (2021). Gene therapies and COVID-19 vaccines: a necessary discussion in relation with viral vector-based approaches. Orphanet J. Rare Dis. *16*, 316.

64. Schulz, M., Levy, D.I., Petropoulos, C.J., Bashirians, G., Winburn, I., Mahn, M., Somanathan, S., Cheng, S.H., and Byrne, B.J. (2023). Binding and neutralizing anti-AAV antibodies: Detection and implications for rAAV-mediated gene therapy. Mol. Ther. *31*, 616–630.

65. Dhungel, B.P., Winburn, I., Pereira, C.D.F., Huang, K., Chhabra, A., and Rasko, J.E.J. (2024). Understanding AAV vector immunogenicity: from particle to patient. Theranostics *14*, 1260–1288.

66. Mendell, J.R., Connolly, A.M., Lehman, K.J., Griffin, D.A., Khan, S.Z., Dharia, S.D., Quintana-Gallardo, L., and Rodino-Klapac, L.R. (2022). Testing preexisting antibodies prior to AAV gene transfer therapy: rationale, lessons and future considerations. Mol. Ther. Methods Clin. Dev. *25*, 74–83.

67. Boutin, S., Monteilhet, V., Veron, P., Leborgne, C., Benveniste, O., Montus, M.F., and Masurier, C. (2010). Prevalence of serum IgG and neutralizing factors against adeno-associated virus (AAV) types 1, 2, 5, 6, 8, and 9 in the healthy population: implications for gene therapy using AAV vectors. Hum. Gene Ther. *21*, 704–712.

68. Calcedo, R., Morizono, H., Wang, L., McCarter, R., He, J., Jones, D., Batshaw, M.L., and Wilson, J.M. (2011). Adeno-associated virus antibody profiles in newborns, children, and adolescents. Clin. Vaccine Immunol. *18*, 1586–1588.

69. Calcedo, R., Vandenberghe, L.H., Gao, G., Lin, J., and Wilson, J.M. (2009). Worldwide epidemiology of neutralizing antibodies to adeno-associated viruses. J. Infect. Dis. *199*, 381–390.

70. Louis Jeune, V., Joergensen, J.A., Hajjar, R.J., and Weber, T. (2013). Pre-existing anti-adeno-associated virus antibodies as a challenge in AAV gene therapy. Hum. Gene Ther. Methods *24*, 59–67.

71. Braun, M., Lange, C., Schatz, P., Long, B., Stanta, J., Gorovits, B., Tarcsa, E., Jawa, V., Yang, T.Y., Lembke, W., et al. (2024). Preexisting antibody assays for gene therapy: Considerations on patient selection cutoffs and companion diagnostic requirements. Mol. Ther. Methods Clin. Dev. *32*, 101217.

72. van Olden, R.W., Lo Bianco, C., Dilly, K.W., Savelieva, M., Xu, S., Tijsma, A., van Baalen, C., Sharma, H., and Mumneh, N. (2024). Adeno-associated virus serotype 9 antibodies in neonates and young children: Seroprevalence and kinetics. Mol. Ther. Methods Clin. Dev. *32*, 101344.

73. Mietzsch, M., Jose, A., Chipman, P., Bhattacharya, N., Daneshparvar, N., McKenna, R., and Agbandje-McKenna, M. (2021). Completion of the AAV Structural Atlas: Serotype Capsid Structures Reveals Clade-Specific Features. Viruses *13*, 101.

74. Yousuf, W., Ibrahim, H., Harfouche, M., Abu Hijleh, F., and Abu-Raddad, L. (2020). Herpes simplex virus type 1 in Europe: systematic review, meta-analyses and meta-regressions. BMJ Glob. Health *5*, e002388.

75. Wang, X., Kerkmann, L., Hetzel, M., Windmann, S., Trilling, M., Zhang, W., Ehrhardt, A., and Bayer, W. (2022). Analysis of the Prevalence of Binding and Neutralizing Antibodies against 39 Human Adenovirus Types

CellPress
OPEN ACCESS

in Student Cohorts Reveals Low-Prevalence Types and a Decline in Binding Antibody Levels during the SARS-CoV-2 Pandemic. J. Virol. *96*, e0113322.

76. Lundstrom, K. (2023). Viral Vectors in Gene Therapy: Where Do We Stand in 2023? Viruses *15*, 698.
77. Dai, Y., Kavita, U., Lampen, M.H., Gielen, S., Banks, G., Levesque, P., Kozhich, A., Pillutla, R., Zhang, Y., Jawa, V., and Adam, L. (2022). Prevalence of Pre-Existing Neutralizing Antibodies Against Adeno-Associated Virus Serotypes 1, 2, 5, 6, 8, and 9 in Sera of Different Pig Strains. Hum. Gene Ther. *33*, 451–459.
78. Rapti, K., Louis-Jeune, V., Kohlbrenner, E., Ishikawa, K., Ladage, D., Zolotukhin, S., Hajjar, R.J., and Weber, T. (2012). Neutralizing antibodies against AAV serotypes 1, 2, 6, and 9 in sera of commonly used animal models. Mol. Ther. *20*, 73–83.
79. Li, P., Boenzli, E., Hofmann-Lehmann, R., and Helfer-Hungerbuehler, A. K. (2019). Pre-existing antibodies to candidate gene therapy vectors (adeno-associated vector serotypes) in domestic cats. PLoS One *14*, e0212811.
80. Delman, K.A., Bennett, J.J., Zager, J.S., Burt, B.M., McAuliffe, P.F., Petrowsky, H., Kooby, D.A., Hawkins, W.G., Horsburgh, B.C., Johnson, P., and Fong, Y. (2000). Effects of preexisting immunity on the response to herpes simplex-based oncolytic viral therapy. Hum. Gene Ther. *11*, 2465–2472.
81. Cao, L., Ledeboer, A., Pan, Y., Lu, Y., and Meyer, K. (2023). Clinical enrollment assay to detect preexisting neutralizing antibodies to AAV6 with demonstrated transgene expression in gene therapy trials. Gene Ther. *30*, 150–159.
82. Ding, J., Murad, Y.M., Sun, Y., Lee, I.F., Samudio, I., Liu, X., Jia, W.W.G., and Zhao, R. (2022). Pre-Existing HSV-1 Immunity Enhances Anticancer Efficacy of a Novel Immune-Stimulating Oncolytic Virus. Viruses *14*, 2327.
83. Lemos de Matos, A., Franco, L.S., and McFadden, G. (2020). Oncolytic Viruses and the Immune System: The Dynamic Duo. Mol. Ther. Methods Clin. Dev. *17*, 349–358.
84. Hui, D.J., Edmonson, S.C., Podsakoff, G.M., Pien, G.C., Ivanciu, L., Camire, R.M., Ertl, H., Mingozzi, F., High, K.A., and Basner-Tschakarjan, E. (2015). AAV capsid CD8+ T-cell epitopes are highly conserved across AAV serotypes. Mol. Ther. Methods Clin. Dev. *2*, 15029.
85. Mingozzi, F., Maus, M.V., Hui, D.J., Sabatino, D.E., Murphy, S.L., Rasko, J.E.J., Ragni, M.V., Manno, C.S., Sommer, J., Jiang, H., et al. (2007). CD8 (+) T-cell responses to adeno-associated virus capsid in humans. Nat. Med. *13*, 419–422.
86. Kuranda, K., Jean-Alphonse, P., Leborgne, C., Hardet, R., Collaud, F., Marmier, S., Costa Verdera, H., Ronzitti, G., Veron, P., and Mingozzi, F. (2018). Exposure to wild-type AAV drives distinct capsid immunity profiles in humans. J. Clin. Investig. *128*, 5267–5279.
87. Li, H., Lasaro, M.O., Jia, B., Lin, S.W., Haut, L.H., High, K.A., and Ertl, H. C.J. (2011). Capsid-specific T-cell responses to natural infections with adeno-associated viruses in humans differ from those of nonhuman primates. Mol. Ther. *19*, 2021–2030.
88. Farber, D.L., Yudanin, N.A., and Restifo, N.P. (2014). Human memory T cells: generation, compartmentalization and homeostasis. Nat. Rev. Immunol. *14*, 24–35.
89. Zhang, W., Yin, Y., Yang, D., Liu, M., Ye, C., Yan, R., and Li, R. (2024). Comprehensive analysis of adverse events associated with onasemnogene abeparvovec (Zolgensma) in spinal muscular atrophy patients: insights from FAERS database. Front. Pharmacol. *15*, 1475884.
90. Li, D., and Wu, M. (2021). Pattern recognition receptors in health and diseases. Signal Transduct. Target. Ther. *6*, 291.
91. Shao, W., Earley, L.F., Chai, Z., Chen, X., Sun, J., He, T., Deng, M., Hirsch, M.L., Ting, J., Samulski, R.J., and Li, C. (2018). Double-stranded RNA innate immune response activation from long-term adeno-associated virus vector transduction. JCI Insight *3*, e120474.
92. Charlesworth, C.T., Hsu, I., Wilkinson, A.C., and Nakauchi, H. (2022). Immunological barriers to haematopoietic stem cell gene therapy. Nat. Rev. Immunol. *22*, 719–733.
93. Kropf, E., Markusic, D.M., Majowicz, A., Mingozzi, F., and Kuranda, K. (2024). Complement System Response to Adeno-Associated Virus Vector Gene Therapy. Hum. Gene Ther. *35*, 425–438.
94. Salabarria, S.M., Corti, M., Coleman, K.E., Wichman, M.B., Berthy, J.A., D'Souza, P., Tifft, C.J., Herzog, R.W., Elder, M.E., Shoemaker, L.R., et al. (2024). Thrombotic microangiopathy following systemic AAV administration is dependent on anti-capsid antibodies. J. Clin. Investig. *134*, e173510.
95. Brandwijk, R.J.M.G.E., Michels, M.A.H.M., van Rossum, M., de Nooijer, A.H., Nilsson, P.H., de Bruin, W.C.C., and Toonen, E.J.M. (2022). Pitfalls in complement analysis: A systematic literature review of assessing complement activation. Front. Immunol. *13*, 1007102.
96. Yang, S., McGookey, M., Wang, Y., Cataland, S.R., and Wu, H.M. (2015). Effect of blood sampling, processing, and storage on the measurement of complement activation biomarkers. Am. J. Clin. Pathol. *143*, 558–565.
97. Ertl, H.C.J. (2021). T Cell-Mediated Immune Responses to AAV and AAV Vectors. Front. Immunol. *12*, 666666.
98. Ronzitti, G., Gross, D.A., and Mingozzi, F. (2020). Human Immune Responses to Adeno-Associated Virus (AAV) Vectors. Front. Immunol. *11*, 670.
99. Duan, D. (2023). Lethal immunotoxicity in high-dose systemic AAV therapy. Mol. Ther. *31*, 3123–3126.
100. Herzog, R.W. (2019). Complexity of immune responses to AAV transgene products - Example of factor IX. Cell. Immunol. *342*, 103658.
101. Shaw, A.R., and Suzuki, M. (2019). Immunology of Adenoviral Vectors in Cancer Therapy. Mol. Ther. Methods Clin. Dev. *15*, 418–429.
102. Kumar, S.R.P., Duan, D., and Herzog, R.W. (2023). Immune Responses to Muscle-Directed Adeno-Associated Viral Gene Transfer in Clinical Studies. Hum. Gene Ther. *34*, 365–371.
103. Bonnemann, C.G., Belluscio, B.A., Braun, S., Morris, C., Singh, T., and Muntoni, F. (2023). Dystrophin Immunity after Gene Therapy for Duchenne's Muscular Dystrophy. N. Engl. J. Med. *388*, 2294–2296.
104. Calcedo, R., Somanathan, S., Qin, Q., Betts, M.R., Rech, A.J., Vonderheide, R.H., Mueller, C., Flotte, T.R., and Wilson, J.M. (2017). Class I-restricted T-cell responses to a polymorphic peptide in a gene therapy clinical trial for alpha-1-antitrypsin deficiency. Proc. Natl. Acad. Sci. USA *114*, 1655–1659.
105. Bali, D.S., Goldstein, J.L., Banugaria, S., Dai, J., Mackey, J., Rehder, C., and Kishnani, P.S. (2012). Predicting cross-reactive immunological material (CRIM) status in Pompe disease using GAA mutations: lessons learned from 10 years of clinical laboratory testing experience. Am. J. Med. Genet. C Semin. Med. Genet. *160C*, 40–49.
106. Valentino, L.A., Ozelo, M.C., Herzog, R.W., Key, N.S., Pishko, A.M., Ragni, M.V., Samelson-Jones, B.J., and Lillicrap, D. (2023). A review of the rationale for gene therapy for hemophilia A with inhibitors: one-shot tolerance and treatment? J. Thromb. Haemost. *21*, 3033–3044.
107. Kishnani, P.S., Dickson, P.I., Muldowney, L., Lee, J.J., Rosenberg, A., Abichandani, R., Bluestone, J.A., Burton, B.K., Dewey, M., Freitas, A., et al. (2016). Immune response to enzyme replacement therapies in lysosomal storage diseases and the role of immune tolerance induction. Mol. Genet. Metab. *117*, 66–83.
108. Khan, A.N., Chowdhury, A., Karulkar, A., Jaiswal, A.K., Banik, A., Asija, S., and Purwar, R. (2022). Immunogenicity of CAR-T Cell Therapeutics: Evidence, Mechanism and Mitigation. Front. Immunol. *13*, 886546.
109. Keeler, G.D., Markusic, D.M., and Hoffman, B.E. (2019). Liver induced transgene tolerance with AAV vectors. Cell. Immunol. *342*, 103728.
110. Kaczmarek, R., Samelson-Jones, B.J., and Herzog, R.W. (2024). Immune tolerance induction by hepatic gene transfer: First-in-human evidence. Mol. Ther. *32*, 863–864.

111. Bluestone, J.A., and Anderson, M. (2020). Tolerance in the Age of Immunotherapy. N. Engl. J. Med. *383*, 1156–1166.

112. Hardet, R., Chevalier, B., Dupaty, L., Naïmi, Y., Riou, G., Drouot, L., Jean, L., Salvetti, A., Boyer, O., and Adriouch, S. (2016). Oral-tolerization Prevents Immune Responses and Improves Transgene Persistence Following Gene Transfer Mediated by Adeno-associated Viral Vector. Mol. Ther. *24*, 87–95.

113. Grygiel-Gorniak, B., Rogacka, N., and Puszczewicz, M. (2018). Antinuclear antibodies in healthy people and non-rheumatic diseases - diagnostic and clinical implications. Reumatologia *56*, 243–248.

114. Xie, N., Shen, G., Gao, W., Huang, Z., Huang, C., and Fu, L. (2023). Neoantigens: promising targets for cancer therapy. Signal Transduct. Target. Ther. *8*, 9.

115. Blass, E., and Ott, P.A. (2021). Advances in the development of personalized neoantigen-based therapeutic cancer vaccines. Nat. Rev. Clin. Oncol. *18*, 215–229.

116. Gianneschi, G., Scolpino, A., and Oleske, J. (2024). A systematic review of the risk of autoimmunity, cancer seeding, and adverse events in human trials of whole-tissue autologous therapeutic vaccines. Cancer Pathog. Ther. *3*, 129–134.

117. Berner, F., and Flatz, L. (2023). Autoimmunity in immune checkpoint inhibitor-induced immune-related adverse events: A focus on autoimmune skin toxicity and pneumonitis. Immunol. Rev. *318*, 37–50.

118. Shen, W., Liu, S., and Ou, L. (2022). rAAV immunogenicity, toxicity, and durability in 255 clinical trials: A meta-analysis. Front. Immunol. *13*, 1001263.

119. Chan, Y.K., Dick, A.D., Hall, S.M., Langmann, T., Scribner, C.L., and Mansfield, B.C.; Ocular Gene Therapy Inflammation Working Group (2021). Ocular Gene Therapy Inflammation Working G. Inflammation in Viral Vector-Mediated Ocular Gene Therapy: A Review and Report From a Workshop Hosted by the Foundation Fighting Blindness, 9/2020. Transl. Vis. Sci. Technol. *10*, 3.

120. Harkins, A.L., Ambegaokar, P.P., and Keeler, A.M. (2024). Immune responses to central nervous system directed adeno-associated virus gene therapy: Does direct CNS delivery make a difference? Neurotherapeutics *21*, e00435.

121. Matthews, E., Brassington, R., Kuntzer, T., Jichi, F., and Manzur, A.Y. (2016). Corticosteroids for the treatment of Duchenne muscular dystrophy. Cochrane Database Syst. Rev. *2016*, CD003725.

122. Russell, D.W., Alexander, I.E., and Miller, A.D. (1995). DNA synthesis and topoisomerase inhibitors increase transduction by adeno-associated virus vectors. Proc. Natl. Acad. Sci. USA *92*, 5719–5723.

123. Cervelli, T., Palacios, J.A., Zentilin, L., Mano, M., Schwartz, R.A., Weitzman, M.D., and Giacca, M. (2008). Processing of recombinant AAV genomes occurs in specific nuclear structures that overlap with foci of DNA-damage-response proteins. J. Cell Sci. *121*, 349–357.

124. Xiang, Z., Kuranda, K., Quinn, W., Chekaoui, A., Ambrose, R., Hasanpourghai, M., Novikov, M., Newman, D., Cole, C., Zhou, X., et al. (2022). The Effect of Rapamycin and Ibrutinib on Antibody Responses to Adeno-Associated Virus Vector-Mediated Gene Transfer. Hum. Gene Ther. *33*, 614–624.

125. Lamming, D.W. (2016). Inhibition of the Mechanistic Target of Rapamycin (mTOR)-Rapamycin and Beyond. Cold Spring Harb. Perspect. Med. *6*, a025924.

126. Monteilhet, V., Saheb, S., Boutin, S., Leborgne, C., Veron, P., Montus, M. F., Moullier, P., Benveniste, O., and Masurier, C. (2011). A 10 patient case report on the impact of plasmapheresis upon neutralizing factors against adeno-associated virus (AAV) types 1, 2, 6, and 8. Mol. Ther. *19*, 2084–2091.

127. Chicoine, L.G., Montgomery, C.L., Bremer, W.G., Shontz, K.M., Griffin, D.A., Heller, K.N., Lewis, S., Malik, V., Grose, W.E., Shilling, C.J., et al. (2014). Plasmapheresis eliminates the negative impact of AAV antibodies on microdystrophin gene expression following vascular delivery. Mol. Ther. *22*, 338–347.

128. Cappell, K.M., and Kochenderfer, J.N. (2023). Long-term outcomes following CAR T cell therapy: what we know so far. Nat. Rev. Clin. Oncol. *20*, 359–371.

129. FDA Center for Biologics Evaluation and Research (CBER). Guidance for Industry: Design and Analysis of Shedding Studies for Virus or Bacteria-Based Gene Therapy and Oncolytic Products; Guidance for Industry Maryland, USA: Food and Drug Administration (FDA); 2015 [Available from: https://www.fda.gov/media/89036/download.

130. Lv, Y., Luo, X., Xie, Z., Qiu, J., Yang, J., Deng, Y., Long, R., Tang, G., Zhang, C., and Zuo, J. (2024). Prospects and challenges of CAR-T cell therapy combined with ICIs. Front. Oncol. *14*, 1368732.

131. Perez-Moreno, M.A., Ciudad-Gutierrez, P., Jaramillo-Ruiz, D., Reguera-Ortega, J.L., Abdel-Kader Martin, L., and Flores-Moreno, S. (2023). Combined or Sequential Treatment with Immune Checkpoint Inhibitors and Car-T Cell Therapies for the Management of Haematological Malignancies: A Systematic Review. Int. J. Mol. Sci. *24*, 14780.

132. Shiravand, Y., Khodadadi, F., Kashani, S.M.A., Hosseini-Fard, S.R., Hosseini, S., Sadeghirad, H., Ladwa, R., O'Byrne, K., and Kulasinghe, A. (2022). Immune Checkpoint Inhibitors in Cancer Therapy. Curr. Oncol. *29*, 3044–3060.

133. Durost, P.A., Aryee, K.E., Manzoor, F., Tisch, R.M., Mueller, C., Jurczyk, A., Shultz, L.D., and Brehm, M.A. (2018). Gene Therapy with an Adeno-Associated Viral Vector Expressing Human Interleukin-2 Alters Immune System Homeostasis in Humanized Mice. Hum. Gene Ther. *29*, 352–365.

134. Nguyen, K.G., Vrabel, M.R., Mantooth, S.M., Hopkins, J.J., Wagner, E.S., Gabaldon, T.A., and Zaharoff, D.A. (2020). Localized Interleukin-12 for Cancer Immunotherapy. Front. Immunol. *11*, 575597.

135. Wang, S.J., Dougan, S.K., and Dougan, M. (2023). Immune mechanisms of toxicity from checkpoint inhibitors. Trends Cancer *9*, 543–553.

136. Kroll, M.H., Rojas-Hernandez, C., and Yee, C. (2022). Hematologic complications of immune checkpoint inhibitors. Blood *139*, 3594–3604.

137. Samaan, M.A., Pavlidis, P., Papa, S., Powell, N., and Irving, P.M. (2018). Gastrointestinal toxicity of immune checkpoint inhibitors: from mechanisms to management. Nat. Rev. Gastroenterol. Hepatol. *15*, 222–234.

138. Gorovits, B., Azadeh, M., Buchlis, G., Harrison, T., Havert, M., Jawa, V., Long, B., McNally, J., Milton, M., Nelson, R., et al. (2021). Evaluation of the Humoral Response to Adeno-Associated Virus-Based Gene Therapy Modalities Using Total Antibody Assays. AAPS J. *23*, 108.

139. Gorovits, B., Fiscella, M., Havert, M., Koren, E., Long, B., Milton, M., and Purushothama, S. (2020). Recommendations for the Development of Cell-Based Anti-Viral Vector Neutralizing Antibody Assays. AAPS J. *22*, 24.

140. Gorovits, B., Marshall, J.C., Smith, J., Whiteley, L.O., and Neubert, H. (2019). Bioanalysis of adeno-associated virus gene therapy therapeutics: regulatory expectations. Bioanalysis *11*, 2011–2024.

141. Pan, L., Mora, J., Walravens, K., Wagner, L., Hopper, S., Loo, L., Bettoun, D., Bond, S., Dessy, F., Downing, S., et al. (2023). White Paper on Recent Issues in Bioanalysis: FDA Draft Guidance on Immunogenicity Information in Prescription Drug Labeling, LNP & Viral Vectors Therapeutics/ Vaccines Immunogenicity, Prolongation Effect, ADA Affinity, Risk-based Approaches, NGS, qPCR, ddPCR Assays (Part 3 - Recommendations on Gene Therapy, Cell Therapy, Vaccines Immunogenicity & Technologies; Immunogenicity & Risk Assessment of Biotherapeutics and Novel Modalities; NAb Assays Integrated Approach). Bioanalysis *15*, 773–814.

142. Mora, J., Palmer, R., Wagner, L., Wu, B., Partridge, M., Meena, S.I., Sonderegger, I., Smeraglia, J., Bivi, N., Dakappagari, N., et al. (2024). White Paper on Recent Issues in Bioanalysis: ISR for ADA Assays, the Rise of dPCR vs qPCR, International Reference Standards for Vaccine Assays, Anti-AAV TAb Post-Dose Assessment, NanoString Validation, ELISpot as Gold Standard (Part 3 - Recommendations on Gene Therapy, Cell Therapy, Vaccines Immunogenicity & Technologies; Biotherapeutics

CellPress
OPEN ACCESS

Immunogenicity & Risk Assessment; ADA/NAb Assay/Reporting Harmonization). Bioanalysis *16*, 77–119.

143. Mora, J.R., White, J.T., Chilewski, S.D., Qu, Q., Stocker, D., Luo, L., and Lavelle, A. (2020). Strategies for method comparison when changes in the immunogenicity method are needed within a clinical program. Bioanalysis *12*, 431–443.

144. Dai, Y., Dong, H., Gleason, C., Mora, J., Kolaitis, G., Balasubramanian, N., Surapaneni, S., Kozhich, A., and Jawa, V. (2023). Comparison of Pre-existing Anti-AAV8 Total Antibody Screening and Confirmatory Assays with a Cell-Based Neutralizing Assay in Normal Human Serum. AAPS J. *25*, 35.

145. Weber, T. (2021). Anti-AAV Antibodies in AAV Gene Therapy: Current Challenges and Possible Solutions. Front. Immunol. *12*, 658399.

146. Liu, D., Ahmet, A., Ward, L., Krishnamoorthy, P., Mandelcorn, E.D., Leigh, R., Brown, J.P., Cohen, A., and Kim, H. (2013). A practical guide to the monitoring and management of the complications of systemic corticosteroid therapy. Allergy Asthma Clin. Immunol. *9*, 30.

147. Kichula, E.A., Proud, C.M., Farrar, M.A., Kwon, J.M., Saito, K., Desguerre, I., and McMillan, H.J. (2021). Expert recommendations and clinical considerations in the use of onasemnogene abeparvovec gene therapy for spinal muscular atrophy. Muscle Nerve *64*, 413–427.

Cell Reports Medicine, Volume *6*

Supplemental information

Current regulatory requirements for assessment of immunogenicity for gene therapy medicinal products

Christopher J. Mann, Jon Giblin, Manuela Braun, Felicitas Schmid, Maria Rathmann Sørensen, Paolo Caferra, Anett Hudák, Tamás Letoha, Núria Coderch, Timothy P. Hickling, and Mimoun Azzouz

Table S1: Summary of current international regulatory guidelines relevant to the assessment of immunogenicity and immunomodulatory regimens for gene therapy medicinal products (GTMPs)

Region (Issuing Agency)	Guideline / Standard	Manufacturing	Nonclinical					Clinical			General		
		Immunogenicity	Animal models	Alternative models	Pre-existing immunity	Treatment responses	Immunomodulation	Pre-existing immunity	Treatment responses	Immunomodulation	Bioanalytical method and CDx	Paediatric	Other
EU / UK (EMA)	Guideline on quality, non-clinical and clinical requirements for investigational advanced therapy medicinal products in clinical trials (EMA/CAT/22473/2025)	-	+	+/-	-	+/-	-	+/-	+/-	-	-	-	-
EU / UK (EMA)	Guideline on the quality, non-clinical and clinical aspects of gene therapy medicinal products (EMA/CAT/80183/2014)	-	+	-	+	+	-	+	+	+/-	-	-	+[1]
EU / UK (EMA)	Reflection paper on quality, non-clinical and clinical issues related to the development of recombinant adeno-associated viral vectors (EMEA/CHMP/GTWP/587488/2007 Rev. 1)	-	+	-	-	-	-	+	+	-	-	-	-
EU / UK (EMA)	Guideline on safety and efficacy follow-up - risk management of advanced therapy medicinal products (Draft) (EMEA/149995/2008 rev.1)	-	-	-	-	-	-	-	+/-	-	-		+[1, 2]
EU / UK (EMA)	Guideline on the risk-based approach according to annex I, part IV of Directive 2001/83/EC applied to Advanced therapy medicinal products	-	-	-	-	+/-	-	-	+/-	-	-	-	+/-[3]

Region (Issuing Agency)	Guideline / Standard	Manufacturing	Nonclinical					Clinical			General		
		Immunogenicity	Animal models	Alternative models	Pre-existing immunity	Treatment responses	Immunomodulation	Pre-existing immunity	Treatment responses	Immunomodulation	Bioanalytical method and CDx	Paediatric	Other
	(EMA/CAT/CPWP/686637 /2011)												
EU / UK (EMA)	Guideline on quality, non-clinical and clinical aspects of medicinal products containing genetically modified cells (EMA/CAT/GTWP/671639 /2008 Rev. 1 – corr)	-	+	+/-	-	-	-	-	+	-	-	-	+/-[1]
EU / UK (EMA)	Guideline on development and manufacture of lentiviral vectors (CHMP/BWP/2458/03)	-	-	-	-	-	-	-	-	-	-	-	-
EU / UK (EMA)	Guidelines on Good Clinical Practice specific to Advanced Therapy Medicinal Products (C(2019) 7140 final)	-	-	-	-	-	-	+/-	+/-	+/-	-	+/-	+/-[4]
USA (FDA)	Chemistry, Manufacturing, and Control (CMC) information for human gene therapy Investigational New Drug Applications (INDs)	-	-	-	-	-	-	-	-	-	-	-	-
USA (FDA)	Long Term Follow-Up after administration of human gene therapy products	-	-	-	-	-	-	-	+/-	+/-	-	-	-
USA (FDA)	Human gene therapy for rare diseases	-	+/-	-	-	-	-	-	+/-	-	+/-	-	-
USA (FDA)	Clinical considerations for therapeutic cancer vaccines	-	-	-	-	-	-	-	+	+	+	-	+[5]

Region (Issuing Agency)	Guideline / Standard	Manufacturing	Nonclinical					Clinical			General		
		Immunogenicity	Animal models	Alternative models	Pre-existing immunity	Treatment responses	Immunomodulation	Pre-existing immunity	Treatment responses	Immunomodulation	Bioanalytical method and CDx	Paediatric	Other
USA (FDA)	Design and analysis of shedding studies for virus or bacteria-based gene therapy and oncolytic products	-	-	-	-	+/-	-	-	-	+/-	-	-	+/-
USA (FDA)	Considerations for the design of early-phase clinical trials of cellular and gene therapy products	-	-	-	-	+/-	-	+	+	+	-	+	+/-[1]
USA (FDA)	Human gene therapy products incorporating human genome editing	-	-	-	-	+	-	+/-	-	-	-	+	-
USA (FDA)	Preclinical assessment of investigational cellular and gene therapy products		+	+	-	+/-	+/-	-	-	-	-	-	-
USA (FDA)	Frequently asked questions - developing potential cellular and gene therapy products	-	+	-	-	-	-	-	-	-	-	-	-
Japan (PMDA)	PMDA (Japan): ensuring the quality and safety of gene therapy products, 9 July, 2019	-	-	-	-	+/-	-	-	+/-	-	-	-	-
Japan (PMDA)	White-paper for quality and safety for gene therapy products using gene editing technology	-	-	-	-	+	-	-	+	-	-	-	+/-[1]

Key concepts related to immunogenicity and immunomodulation that were identified as being the most relevant in the landscape assessment are shown at the top. Each guideline is qualified for relative coverage of these key areas with "-" indication no or negligible coverage, "+/-" some coverage and "+" indication that the key area is addressed.

[1] Refers to risk of autoimmunity (including possibly reference to cross-reactivity or bystander autoimmune responses).

[2] Specific reference to risk management plan (RMP).

[3] Discussion on localisation of relevant studies and assessment in regulatory documentation.

[4] Discussion of specific pharmacovigilance aspects.

[5] Discussion on use of adjuvants in addition to therapeutic agent (and independently of other immunomodulatory agents)

Table S2: List of additional guidelines and monographs not specific to GTMPs but relevant to evaluation of immunogenicity to therapeutic proteins and biologics and/or developing companion diagnostics (CDx)

**Biologic product guidance**	
EU /EEA	EMA Guideline on Immunogenicity assessment of therapeutic proteins, Rev 1, 2017 (EMEA/CHMP/BMWP/14327/2006 Rev 1)
USA	FDA Immunogenicity Assessment for Therapeutic Protein Products, 2104
USA	FDA Immunogenicity Testing of Therapeutic Protein Products —Developing and Validating Assays for Anti-Drug Antibody Detection, 2019
**Guidance/legislation relevant to bioanalytical methods, companion diagnostics and/or patient stratification**	
EU /EEA	Regulation (EU) 2017/746 of the European parliament and of the council of 5 April 2017 on in vitro diagnostic medical devices and repealing Directive 98/79/EC and Commission Decision 2010/227/EU
USA	FDA In Vitro Companion Diagnostic Devices, 2014
Other / Rest of the world (RoW)	ICH M10 Bioanalytical method validation and study sample analysis<br>CLSI EP24-A2 Assessment of the Diagnostic Accuracy of Laboratory Tests Using Receiver Operating Characteristic Curves; Approved Guideline – Second Edition<br>CLSI EP28-A3c Defining, Establishing, and Verifying Reference Intervals in the Clinical Laboratory; Approved Guideline – Third Edition<br>CLSI EP12-A2 User Protocol for Evaluation of Qualitative Test Performance; Approved Guideline – Second Edition<br>CLSI EP17-A Protocols for Determination of Limits of Detection and Limits of Quantitation. Approved Guideline<br>CLSI EP17-A2 Evaluation of Detection Capability for Clinical Laboratory Measurement Procedures; Approved Guideline – Second Edition
International Standards Organisation (ISO)	MD ISOs for combined ATMPs - ISO/TS 10993-20:2006(en) Biological evaluation of medical devices — Part 20: Principles and methods for immunotoxicology testing of medical devices
Key papers	Evaluation of Cellular Immune Response to Adeno-Associated Virus-Based Gene Therapy (Gorovits *et al*., 2023)<br>Evaluation of the Humoral Response to Adeno-Associated Virus-Based Gene Therapy Modalities Using Total Antibody Assays (Gorovits *et al*., 2021)<br>Bioanalysis of adeno-associated virus gene therapy therapeutics: regulatory expectations (Gorovits *et al*., 2020)<br>2023 White Paper on Recent Issues in Bioanalysis: ISR for ADA Assays, the Rise of dPCR vs qPCR, International Reference Standards for Vaccine Assays, Anti-AAV TAb Post-Dose Assessment, NanoString Validation, ELISpot as Gold Standard (Part 3 - Recommendations on Gene Therapy, Cell Therapy, Vaccines Immunogenicity & Technologies; Biotherapeutics Immunogenicity & Risk Assessment; ADA/NAb Assay/Reporting Harmonization (Mora et al., 2024)<br>2022 White Paper on Recent Issues in Bioanalysis: FDA Draft Guidance on Immunogenicity Information in Prescription Drug Labeling, LNP & Viral Vectors Therapeutics/Vaccines Immunogenicity, Prolongation Effect, ADA Affinity, Risk-based Approaches, NGS, qPCR, ddPCR Assays (Part 3 - Recommendations on Gene Therapy, Cell Therapy, Vaccines Immunogenicity & Technologies; Immunogenicity & Risk Assessment of Biotherapeutics and Novel Modalities; NAb Assays Integrated Approach) (Pan et al., 2023)

Definitions:

Autoimmunity: an immune response against an individual's own cells, tissues, or components, potentially leading to disease.

Bystander activation: antigen-independent activation of self-reactive B and T cells that may promote autoimmunity.

Companion diagnostic (CDx): an in vitro diagnostic test that supports the safe and effective use of a specific corresponding medicinal product, for example, by identifying patients that are suitable or unsuitable for treatment such as those with critical levels of pre-existing immunity (Regulation 2017/746/EU).

Cross-reactivity: when an antigen elicits immune responses that cross react with other antigens.

Immunogenicity: ability of an agent to generate immune response against itself.

Immunomodulation: regulation and modulation of immunity by agents that enhance or suppress immunological function.

Pharmacovigilance: the science and activities relating to the detection, assessment, understanding, and prevention of adverse effects or any other drug-related problem.
